# Genome-Wide Identification and Expression Analysis of *Sucrose Phosphate Synthase*, *Sucrose Synthase*, and *Invertase* in Carrot (*Daucus carota* L.)

**DOI:** 10.3390/genes17101184

**Published:** 2026-09-24

**Authors:** Zhen Shi, Xiaoping Kong, Hao Sun, Xiangping Yan, Tingzhen Wei, Qiwen Zhong

**Affiliations:** 1 College of Agriculture and Animal Husbandry, Qinghai University, Xining 810016, China; szzssl@mailbox.qhu.edu.cn; 2 Xining Botanical Garden, Xining 810003, China; 3 Henan Key Laboratory of Ion-Beam Green Agriculture Bioengineering, School of Agriculture and Biomanufacturing, Zhengzhou University, Zhengzhou 450001, China; sunhau@zzu.edu.cn; 4 Xining Vegetable Technical Service Center, Xining 810003, China; xiangping09@163.com (X.Y.); kkgg_2221@163.com (T.W.); 5 Academy of Agriculture and Forestry Sciences, Qinghai University, Xining 810016, China

**Keywords:** carrot, sucrose metabolism, gene family, abiotic stress, expression profiling

## Abstract

Background: Carbon partitioning and energy supply in plants are largely dictated by sucrose metabolism, which is coordinately mediated by sucrose phosphate synthase (SPS), sucrose synthase (SUS), and invertase (INV). Carrot is an economically important root crop worldwide. As the major transport and storage disaccharide, sucrose modulates root osmotic potential and sweetness depending on its accumulation level. However, the genome-wide characteristics of the *SPS*, *SUS*, and *INV* gene families in carrot, as well as their expression dynamics across developmental stages and under stress conditions, remain unknown. Methods: In this study, we identified these gene families in carrot and characterized their phylogenetic relationships, gene structures, conserved protein motifs, chromosomal distribution, and expression profiles across developmental stages and under abiotic treatment with stress-inducing agents. Results: A total of 5 *SPS*, 8 *SUS*, and 19 *INV* genes were identified in carrot. Phylogenetic analysis revealed that members within the same subfamily share similar exon–intron structures and conserved motif compositions. Segment duplication, rather than tandem duplication, emerged as the primary driver of family expansion. Transcript profiling revealed that *DcSPS*, *DcSUS*, and *DcINV* paralogs exhibit varied spatiotemporal expression signatures across tissues and developmental stages, and several are robustly activated as fleshy roots expand. Upon abiotic challenge, these genes undergo differential transcriptional modulation, and many members show pronounced induction under NaCl or exogenous ABA treatment. Conclusions: Collectively, these findings provide a comprehensive inventory of the *SPS*, *SUS*, and *INV* gene families in carrot and generate testable hypotheses regarding their potential roles in sucrose metabolism. The identified genes and their expression profiles offer a valuable genetic resource for future functional studies and sucrose-based quality improvement in this crop.

## 1. Introduction

Carrot (*Daucus carota* L.) is a globally cultivated root vegetable with outstanding nutritional value. Its fleshy taproots accumulate abundant bioactive metabolites, including carotenoids, vitamins, and indispensable mineral elements; these compounds endow carrots with strong antioxidant capacity and confer multiple human health-promoting properties [1]. Soluble sugars represent core primary metabolites stored within carrot taproots. Beyond governing the characteristic sweet flavor of carrot flesh, sugar accumulation substantially modulates overall root nutritional quality and dictates the crop’s commercial market performance.

Sucrose is the primary transport form of photosynthetically assimilated carbon in higher plants [2]. In addition to its fundamental role in energy metabolism, sucrose functions as a critical signaling molecule that governs plant growth, development, organ differentiation, and stress responses [3,4]. In higher plants, sucrose metabolism is coordinately regulated by key enzyme families, including sucrose phosphate synthase (SPS), sucrose synthase (SUS), and invertase (INV). Among these, SPS serves as the core enzyme in sucrose synthesis, catalyzing the conversion of uridine diphosphate glucose (UDPG) and fructose-6-phosphate (F6P) into sucrose-6-phosphate (S6P), which is subsequently dephosphorylated to sucrose by sucrose-6-phosphate phosphatase (SPP). SPS and SPP function synergistically in sucrose biosynthesis. The activity levels of SPS and SUS are closely related to sucrose accumulation and serve as important indicators of a plant’s ability to synthesize and catabolize sucrose [5]. SUS catalyzes a reversible reaction between sucrose and uridine diphosphate (UDP) to produce UDPG and fructose [6], representing a critical pathway for both sucrose catabolism and the generation of glucosyl donors. The primary function of SUS is to mediate the enzymatic cleavage of sucrose within the symplastic pathway, thereby regulating the effective unloading of photosynthetic products between source and sink tissues and facilitating the redistribution of carbon sources. INVs are ubiquitously present in plants and play crucial roles in key physiological processes, including the regulation of sugar metabolism, growth and development, and responses to abiotic stress [7]. INV can be classified into acidic invertases (AINVs) and alkaline / neutral invertases (NINVs) based on their optimal pH levels [8]. AINVs exhibit an optimal pH range of 4.5–5.0, belong to glycoside hydrolase family 32 (GH32), and are typically localized in the cell wall or vacuole [9]. In contrast, NINVs have an optimal pH range of 6.5–8.0, belong to glycoside hydrolase family 100 (GH100), and are primarily localized in the cytoplasm, mitochondria, or plastids [10].

Studies in plants such as tomato have shown that *SPS*, *SUS*, and *INV* are all encoded by polygenic families [11]. To date, 4, 5, and 6 *SPS* members have been identified in Arabidopsis [12], rice [13], and apple [14], respectively. Based on phylogenetic analysis of amino acid sequences, the SPS protein family has been divided into three subfamilies: A, B, and C. Members of subfamily A play a dominant role in sucrose synthesis, and subsequent studies have identified a D subfamily specific to grass species [15]. The number of members in the *SUS* gene family varies significantly among species: there are 6 in both Arabidopsis [16] and rice [17], 3 in maize [18], and as many as 15 in poplar [19]. Phylogenetic analysis has classified them into three subfamilies: *SUSI*, *SUSII*, and *SUSIII*. Early work provided the first molecular characterization of sucrose-cleaving enzymes in carrot, isolating cDNA clones for cell wall invertase, vacuolar invertase isoenzymes I and II, and sucrose synthase, and revealing organ- and development-specific expression patterns [20]. This study laid the foundation for subsequent research on sucrose-cleaving enzymes in carrot. To date, only *DcSUS1*, *DcSUS2*, and *DcSUS3* have been identified in carrots [21]; the identification of other family members remains unclear. *Arabidopsis* and tomato serve as well-characterized eudicot model species, whereas rice represents a monocot reference whose sugar storage strategy differs substantially from that of carrot. In Arabidopsis and rice, 8 *AINVs* and 2 *NINVs* and 11 *AINVs* and 5 *NINVs*, respectively, have been identified [22], while in tomato, 11 *AINVs* and 8 *NINVs* have been identified [11]. However, the structural characteristics of *INV* genes in carrots have not been comprehensively analyzed.

SPS, SUS, and INV are essential for organogenesis, growth, development, and stress responses in plants. As the key rate-limiting enzyme in sucrose synthesis, SPS shows a degree of functional conservation across various crops. For example, genome-wide analysis has revealed that cassava contains 5 *SPS* genes, which are distributed across 5 chromosomes. These genes demonstrate spatiotemporal and tissue-specific expression patterns, with predominant expression observed in leaves. Furthermore, their expression levels show a positive correlation with starch content in the storage roots [23]. Similar results were observed in cotton overexpressing the spinach *SPS* gene [24], indicating that enhanced SPS activity can directly facilitate the structural growth of sink organs. SUS is a key enzyme in sucrose catabolism, and the expansion and functional diversification of its family members are crucial for crop yield. In sugar beet, *SBSS2* is more highly expressed in young storage roots than *SBSS1*, suggesting that it may be involved in carbon supply during the early stages of storage organ development [25]. Heterologous expression of poplar *PsnSuSy2* in tobacco elevates cellulose deposition, thickens cell walls and boosts plant stature [26]; this further confirms the conserved role of SUS in structural development. INV is a crucial enzyme that catalyzes the irreversible hydrolysis of sucrose. Transcriptomic analyses of wheat indicate that *INV* gene members display diverse and distinct expression patterns across various tissues, including roots, stems, leaves, spikes, and grains [27]. Cumulative studies further reveal that INV participates in the regulation of sucrose partitioning, as well as seed/pollen development, floral and fruit growth, and stem and root morphogenesis [7,27,28,29]. Additionally, SPS, SUS, and INV have also been implicated in plant responses to abiotic stress. For instance, under drought stress, the expression levels of *SlSPS1*, *SlCIN3*, *SlVIN2*, and *SlCWIN2* in tomatoes were significantly upregulated. This water deficit appears to promote sucrose accumulation by modulating the expression of genes related to sucrose metabolism in fruits [30]. Therefore, understanding the response patterns of these genes under stress conditions is crucial for enhancing stress tolerance and quality in crops.

Characterization of carrot *SPS*, *SUS* and *INV* gene families facilitates exploration of sugar metabolism and accumulation in carrot. Nevertheless, systematic information regarding these three gene families in carrot remains largely unreported. In this study, we performed genome-wide identification of the carrot *SPS*, *SUS*, and *INV* gene families and characterized their chromosomal localization, phylogenetic relationships, gene structures, conserved domains, and promoter cis-acting elements. In addition, their expression profiles across different developmental stages and under selected abiotic treatments with stress-inducing agents were also investigated. These findings provide candidate genes and a basis for future functional studies of *DcSPS*, *DcSUS*, and *DcINV* and may serve as valuable genetic resources for stress tolerance and quality improvement in carrot breeding programs.

## 2. Materials and Methods

### 2.1. Identification of the SPS, SUS, and INV in Carrot

To identify SPS, SUS, and INV in carrot, the genome sequence, protein sequence, and genome position annotation files were downloaded from the NCBI database (https://www.ncbi.nlm.nih.gov/datasets/genome/, accessed on 20 August 2025). Hidden Markov Model (HMM) profiles for the conserved domains of each gene family were obtained from the Pfam database (https://www.ebi.ac.uk/interpro/entry/pfam/, accessed on 21 August 2025). These included the Pfam (Protein Families database) accession numbers PF00534, PF00862, and PF05116 for the SPS family [31]; PF00534 and PF00862 for the SUS family [31]; and NINV:PF12899, CWINV:PF08244 and PF00251, and VINV:PF11837, PF08244, and PF00251 for the INV family [32]. Based on these HMM profiles, a preliminary screening of the carrot protein sequences was conducted using the Simple HMM Search tool in TBtools-II v2.322, with an E-value threshold of 1 × 10^−5^. Concurrently, BLASTP searches in TBtools-II v2.322 were performed against the carrot protein database using the previously identified Arabidopsis SPS, SUS, and INV protein sequences as queries to retrieve carrot homologs. The Arabidopsis reference sequences used were as follows: for SPS, *At5g20280* (*AtSPS1F*), *At5g11110* (*AtSPS2F*), *At1g04920* (*AtSPS3F*), and *At4g10120* (*AtSPS4F*); for SUS, *At5g20830* (*AtSUS1*), *At5g49410* (*AtSUS2*), *At4g02280* (*AtSUS3*), *At3g43190* (*AtSUS4*), *At5g37180* (*AtSUS5*), and *At1g73370* (*AtSUS6*); and for INV, *At3g52600* (*AtCWINV2*), *At2g36190* (*AtCWINV4*), *At3g13784* (*AtCWINV5*), *At5g11920* (*AtCWINV6*), *At1g55120* (*AtFRUCT5*), *At3g13790* (*AtBFRUCT1*), *At1g62660*, *At1g12240* (*AtATVI2*), *At1g56560* (*AtAN-INVA*), *At4g34860* (*AtAN-INVB*), *At3g06500* (*AtAN-INVC*), *At1g35580* (*AtAN-INVG*), *At4g09510* (*AtAN-INV1*), *At1g22650* (*AtAN-INVD*), *At1g72000* (*AtAN-INVF*), *At5g22510* (*AtAN-INVE*), and *At3g05820* (*AtAN-INVH*) [11]. Candidate genes were identified by integrating results from HMM screening and BLASTP searches in TBtools-II v2.322. The integrity of the conserved domains in the candidate proteins was verified using the NCBI-CDD (https://www.ncbi.nlm.nih.gov/Structure/bwrpsb/bwrpsb.cgi, accessed on 1 September 2025) and InterPro (https://www.ebi.ac.uk/interpro/search/sequence/, accessed on 2 September 2025) databases. Sequences with incomplete domains or identified as false positives were discarded to establish the final set of family members. The sequence length, molecular weight, and isoelectric point of the identified proteins were analyzed using the ExPASy online tool (https://www.expasy.org/, accessed on 3 September 2025). The subcellular localization of the DcSPS, DcSUS, and DcINV proteins was predicted using WoLF PSORT (https://wolfpsort.hgc.jp/, accessed on 4 September 2025).

### 2.2. Analysis of Phylogenetic Relationships, Gene Structure, Conservative Motifs, and Protein Domains

Genome sequences, protein sequences, and genome annotation files for Arabidopsis thaliana and rice were downloaded from the Ensembl Plants database (https://plants.ensembl.org/index.html, accessed on 10 September 2025). Multiple sequence alignments were performed using the ClustalW tool in MEGA-X v11.0.13, and a phylogenetic tree was constructed using the maximum likelihood method [33]. Gene structures of the *DcSPS*, *DcSUS*, and *DcINV* gene family members were analyzed using TBtools-II v2.322 [34]. Conserved motifs in the proteins of each family were identified using the online tool MEME (https://meme-suite.org/meme/tools/meme, accessed on 15 September 2025), with the number of motifs to search set to 10 [35]. Protein conserved domains were analyzed using the NCBI BATCH CD-Search tool (https://www.ncbi.nlm.nih.gov/Structure/bwrpsb/bwrpsb.cgi, accessed on 11 September 2025) in conjunction with the Pfam database. Finally, the gene structures, conserved motifs, and conserved domains were integrated and visualized using TBtools-II v2.322.

### 2.3. Chromosomal Localization, Synteny, and Gene Duplication Analysis

Based on the carrot genome and annotation files, the chromosomal localization of SPS, SUS, and INV gene family members was visualized using the Gene Location Visualize tool in TBtools-II v2.322. To explore the interspecific syntenic relationships between carrot and Arabidopsis thaliana as well as rice, synteny analysis was performed using TBtools-II v2.322 and MCScanX [36] to identify gene duplication events. The syntenic relationships among members of the *DcSPS*, *DcSUS*, and *DcINV* families were then visualized using the Advanced Circos tool in TBtools-II v2.322. Furthermore, the Ka/Ks ratios for duplicated gene pairs were calculated using the Simple Ka/Ks Calculator (NG) tool in Tbtools to evaluate the selection pressure acting during their evolutionary history.

### 2.4. Cis-Regulatory Element and Tissue-Specific Expression Analysis

Promoter sequences spanning 2000 bp upstream of the transcription start sites of the *DcSPS*, *DcSUS*, and *DcINV* genes were extracted. Cis-acting regulatory elements in these promoter regions were predicted using the Plant-CARE database (http://bioinformatics.psb.ugent.be/webtools/plantcare/html/, accessed on 3 October 2025) [37]. To analyze the expression profiles of each gene family member in different carrot tissues, publicly available RNA-seq data for carrots were obtained from the NCBI SRA database [38]. The RNA-seq data analyzed in this study are available in the NCBI SRA repository (https://www.ncbi.nlm.nih.gov/sra/, accessed on 11 September 2025), accession number SRP062159. Expression values were calculated as log_2_(TPM + 1), where TPM represents transcripts per million.

### 2.5. Plant Materials, Growth Conditions, and Treatment with Stress-Inducing Agents

In this study, two carrot cultivars, ‘Sanhong’ and ‘Yizhila’, were utilized as experimental materials. The plants were cultivated in a controlled-environment growth chamber with a photoperiod of 16 h light and 8 h dark at a temperature of 25 °C and a relative humidity of 50–70%. ‘Sanhong’ was used for developmental expression profiling, with fleshy roots and leaves collected at 60, 80, 100, and 120 days post-sowing. Conversely, ‘Yizhila’ was subjected to treatment with stress-inducing agents; 60-day-old plants were treated with 150 mM NaCl as a stress treatment [39], or with 50 µM ABA as an inducer of stress-mediated signaling [11], and root and leaf tissues were harvested at 0, 6, 12, 24, and 72 h post-treatment. Untreated control plants were sampled only at 0 h. The samples were snap-frozen in liquid nitrogen and stored at −80 °C for subsequent RNA isolation.

### 2.6. RNA Extraction, Reverse Transcription, and Quantitative Real-Time PCR Analysis

Total RNA was extracted from root and leaf tissues using the E.Z.N.A.^®^ Plant RNA Kit (Omega Bio-tek, Norcross, GA, USA; Catalog: R6827-01) according to the manufacturer’s instructions. The extracted RNA was reverse transcribed into complementary DNA (cDNA) utilizing the EX RT Kit (with gDNA remover) (ZOMANBIO, Beijing, China; Catalog: #ZR108). Quantitative real-time PCR (qRT-PCR) was conducted on an Eppendorf Mastercycler^®^ ep realplex 2 real-time PCR system (Eppendorf, Hamburg, Germany). The thermal cycling protocol consisted of an initial denaturation step at 95 °C for 30 s, followed by 40 cycles of denaturation at 95 °C for 10 s and annealing/extension at 60 °C for 30 s. Melt curve analysis was performed using the default thermal cycling program of the instrument. The 20 µL reaction mixture included 10 µL of 2× HQ SYBR qPCR Mix (without ROX) (ZOMANBIO, Beijing, China; Catalog: #ZF501), 2 µL of cDNA template, 0.4 µL each of forward and reverse primers, and 7.2 µL of nuclease-free ddH_2_O. *Actin* [40,41] and *5.8S rRNA* [42] were utilized as internal reference genes, and relative gene expression was calculated using the 2^−ΔΔCt^ method [43]. In this study, quantitative polymerase chain reaction (qPCR) experiments were performed using three biological replicates, with each sample consisting of three technical replicates. The sequences of the primers are presented in Table S5.

## 3. Results

### 3.1. Identification of the SPS, SUS, and INV Genes in Carrots

Utilizing a combined screening approach that incorporates BLAST and HMMER as integrated in TBtools-II v2.322, followed by validation against the NCBI-CDD database, we identified a total of 5 *SPS*, 8 *SUS*, and 19 *INV* genes within the carrot genome. Based on their chromosomal locations, these genes were designated as *DcSPS1*–*DcSPS5*, *DcSUS1*–*DcSUS8*, *DcINVCW1*–*DcINVCW10*, *DcINVVR1*–*DcINVVR3*, and *DcINVAN1*–*DcINVAN6* (Table 1).

The five identified *DcSPS* genes in carrot exhibit coding sequences (CDSs) ranging from 3153 to 3225 bp, which encode proteins comprising 1050 to 1074 amino acids. These proteins have molecular weights varying from 118.14 to 120.67 kDa and isoelectric points (pI) between 5.7 and 7.0. The instability indices of the 5 DcSPS proteins range from 45.90 to 49.02, all exceeding the threshold of 40, thereby classifying them as unstable proteins. The grand average of hydropathicity (GRAVY) values falls between −0.470 and −0.353, with all values being below zero, indicating that all DcSPS proteins are hydrophilic. Predictions regarding subcellular localization suggest that *DcSPS1*, *DcSPS3*, and *DcSPS4* are likely localized in the nucleus, while *DcSPS2* is predicted to be found in the chloroplast and *DcSPS5* in the cytoplasm.

In the carrot genome, eight *DcSUS* genes were identified, with their coding sequences (CDSs) ranging in length from 2406 to 2658 bp. These genes encode proteins that vary in size from 801 to 885 amino acids, with corresponding molecular weights between 91.65 and 100.32 kDa and isoelectric points (pIs) ranging from 5.85 to 6.94. The instability indices of these eight DcSUS proteins ranged from 33.31 to 40.03; notably, only *DcSUS5* exhibited an instability index exceeding 40, indicating that the majority of DcSUS proteins are classified as stable. The grand average of hydropathicity (GRAVY) values ranged from −0.313 to −0.192, all of which are below zero, suggesting that all DcSUS proteins are hydrophilic. Predictions for subcellular localization indicated that, except for *DcSUS3*, which is predicted to localize to the nucleus, and *DcSUS4*, predicted to localize to the chloroplast, most DcSUS proteins are likely localized to the cytoplasm.

In the carrot genome, a total of 19 *INV* genes were identified, which include 13 *AINVs* and 6 *NINVs*. Based on their conserved domains, the acid invertase genes were further classified into cell wall invertase genes (*DcINVCW1*–*DcINVCW10*) and vacuolar invertase genes (*DcINVVR1*–*DcINVVR3*). The members of the DcINVCW family exhibited CDS lengths ranging from 1392 to 1914 bp, encoding proteins comprising 463 to 637 amino acids, with molecular weights from 51.52 to 70.93 kDa and isoelectric points (pIs) between 4.52 and 8.99. Their instability indices varied from 25.23 to 38.39, all below 40, indicating that these ten proteins are classified as stable. The grand average of hydropathicity (GRAVY) values ranged from −0.43 to −0.198, all below zero, suggesting that all DcINVCW proteins are hydrophilic. Predictions of subcellular localization indicated that these proteins are distributed in the chloroplast, nucleus, and cytoplasm. For the DcINVVR family, the CDS lengths ranged from 1956 to 2010 bp, encoding proteins of 651 to 669 amino acids, with molecular weights between 72.71 and 74.97 kDa and isoelectric points from 5.05 to 5.8. Their instability indices ranged from 32.76 to 39.65, and the GRAVY values varied from −0.346 to −0.260, all below zero, indicating that they are also hydrophilic proteins.

The DcINVAN family members exhibited considerable variation in coding sequence (CDS) length and amino acid count. Their CDS lengths ranged from 1674 to 2028 bp, encoding proteins comprising 557 to 675 amino acids, with molecular weights ranging from 63.63 to 76.10 kDa and isoelectric points (pIs) from 5.97 to 8.26. The instability indices varied from 37.30 to 58.97, with *DcINVAN2* and *DcINVAN4* demonstrating values below 40, classifying these two proteins as stable. The grand average of hydropathicity (GRAVY) values ranged from −0.367 to −0.229, all being negative, which indicates that all DcINVAN proteins are hydrophilic. Subcellular localization predictions revealed that *DcINVAN1*, *DcINVAN5*, and *DcINVAN6* are localized to the cytoplasm, while *DcINVAN2* and *DcINVAN4* are localized to the chloroplast, and *DcINVAN3* is found in the nucleus.

### 3.2. Phylogenetic Analysis of the SPS, SUS, and INV Families

In this study, the MEGA-X v11.0.13 software was utilized to construct phylogenetic trees of SPS, SUS, and INV proteins derived from carrots, Arabidopsis, and rice, employing the maximum likelihood method. The SPS family is categorized into four subfamilies—A, B, C, and D—based on the phylogenetic analysis. DcSPS1, DcSPS3, and DcSPS4 are phylogenetically closely related to AtSPS1F and AtSPS2F from Arabidopsis, clustering together in subfamily A. In contrast, DcSPS5 clusters with AtSPS3F and OsSPS1 in subfamily B. Additionally, DcSPS2, AtSPS4F, and OsSPS11 are grouped in subfamily C, while subfamily D consists solely of specific SPS members from rice (Figure 1A). This observation aligns with previous research indicating that subfamily D is exclusively found within the Poaceae family [15].

The analysis of the evolutionary relationships among SUS family genes from carrot, Arabidopsis thaliana, and rice has revealed that these proteins can be classified into three distinct subfamilies: SUSI, SUSII, and SUSIII (Figure 1B). The proteins DcSUS1, DcSUS7, and DcSUS8 exhibit a close phylogenetic affinity with Arabidopsis AtSUS1 and AtSUS4, clustering within the SUSI subfamily. Within the SUSII subfamily, DcSUS2 and DcSUS5 form a clade with AtSUS3 and OsSUS4, while DcSUS4 clusters with AtSUS2 in a separate clade; together, these two clades constitute the SUSII subfamily. In the SUSIII subfamily, DcSUS6 groups with AtSUS5, and DcSUS3 clusters with AtSUS6, indicating that members of this subfamily are more closely related to their Arabidopsis counterparts (Figure 1B).

To investigate the phylogenetic relationships among invertase proteins in carrot, Arabidopsis thaliana, and rice, we constructed phylogenetic trees separately for the AINV and VINV proteins of the three species. The results revealed that DcINVCW1, DcINVCW4, DcINVCW6, DcINVCW7, DcINVCW9, and DcINVCW10 clustered with selected cell wall invertases from Arabidopsis and rice, forming a distinct clade designated as the cell wall invertase subfamily. In contrast, DcINVVR1, DcINVVR2, and DcINVVR3 were grouped within a vacuolar invertase clade alongside Arabidopsis genes At1g62660 and At1g12240 (AtATVI2) and rice genes OsINV3 and OsINV2, indicating a close phylogenetic affinity. Notably, despite being annotated as cell wall invertases, DcINVCW2, DcINVCW3, DcINVCW5, and DcINVCW8 were phylogenetically positioned within the vacuolar invertase clade (Figure 1C). This finding may be attributed to the presence of tandemly duplicated gene pairs and the high sequence similarity of these genes with DcINVVR1 and DcINVVR3.

Previous studies have demonstrated that INVAN proteins localized to various subcellular compartments exhibit distinct functional specializations in plant sugar transport and metabolism. Specifically, cytosolic INVAN proteins are responsible for sucrose hydrolysis in the cytosol [44], plastidic INVAN proteins participate in chloroplast sugar metabolism [45], and mitochondrial INVAN proteins supply energy for mitochondrial activities, with this functional differentiation pattern being evolutionarily conserved [46]. In this study, a phylogenetic analysis of A/N-type invertases from carrot, Arabidopsis, and rice revealed that NINVs from these species can be categorized into two major subfamilies, designated α and β. The α subfamily was further subdivided into two clades, α1 and α2, based on predicted subcellular localization. Members of the carrot DcINVAN family were distributed across these evolutionary clades: DcINVAN1, DcINVAN3, and DcINVAN6 were assigned to the β subfamily; DcINVAN5 belonged to the α1 clade; and DcINVAN2 and DcINVAN4 were placed in the α2 clade. Most carrot DcINVAN genes formed well-supported clades with their Arabidopsis orthologs. Notably, Arabidopsis AtAN-INVG and AtAN-INVI clustered preferentially together before forming a clade with DcINVAN1, suggesting a closer phylogenetic relationship among cytosolic INVAN from Arabidopsis.

### 3.3. Conserved Domains, Conserved Motifs, and Gene Structures of the SPS, SUS, and INV Gene Families

The analysis of the carrot *SPS* gene family revealed that all SPS members contain the Glycos_transf_1 (PF00534), Sucrose_synth (PF00862), and S6PP (PF05116) domains (Figure 2A). Among these, Glycos_transf_1 is part of the GT-B-type glycosyltransferase superfamily (Cl10013) [47] and serves as the core catalytic module mediating the transfer of glycosyl groups. This module is responsible for the directed transfer of glucose donors to acceptor molecules, providing the essential catalytic activity for sucrose-6-phosphate synthesis. The S6PP domain may function as a specific binding site for SPP within the SPS protein [48], facilitating the formation of an ‘SPS–SPP’ catalytic complex that coordinately accomplishes sucrose synthesis. Conserved motif analysis predicted ten conserved motifs, each occurring only once in each sequence. The analysis of the positions of domains and motifs revealed that motifs 4, 8, and 5 belonged to the Sucrose_synth domain, motif 2 belonged to the Glycos_transf_1 domain, and motif 10 belonged to the S6PP domain. Gene structure analysis indicated that members of the same subfamily share similar intron–exon numbers and distribution patterns. Notably, *DcSPS4*, *DcSPS1*, and *DcSPS3* each contain 13 introns and 14 exons, suggesting that these structural features are evolutionarily conserved. Further analysis revealed that *DcSPS5* exhibits deletions of exon fragments, specifically a deletion of exon 2, while *DcSPS2* shows an insertion of an exon within the intron 4 region. Such structural variations may provide a basis for functional divergence within the carrot *SPS* gene family.

The analysis of the carrot *SUS* gene family revealed that all eight *SUS* genes in carrot contain two typical domains: Sucrose_synth (PF00862) and Glycos_transf_1 (PF00534) (Figure 2B). Using the MEME website, ten conserved motifs were predicted in the SUS proteins, with each motif occurring only once in each sequence. Among these, motifs 10, 9, 5, 4, 3, and 2 belonged to the Sucrose_synth domain, while motif 1 belonged to the Glycos_transf_1 domain. Analysis of the intron–exon structures of the *DcSUS* genes revealed that members within the same subfamily share similar numbers and distribution patterns of introns and exons. Notably, *DcSUS7*, *DcSUS8*, and *DcSUS1* exhibit identical intron and exon numbers, suggesting a relatively conserved intron–exon structure among these subfamily members. Further analysis indicated structural divergence in the intron–exon organization of *DcSUS4* and *DcSUS6*. Specifically, an exon insertion was detected in the first intron of *DcSUS4*, while the first exon of *DcSUS6* was found to be longer than that of other members, likely due to the elongation of its first intron. These observations suggest that the intron–exon structures of *DcSUS4* and *DcSUS6* have undergone significant structural alterations.

The analysis of the carrot *INV* family indicates that the Glyco-hydro-32N and Glyco-hydro-32C domains are present in all DcINVVR proteins, while the DUF3357 domain is exclusive to DcINVVR (Figure 2C). A detailed examination of conserved motifs reveals that *DcINVCW3* is missing motifs 3 and 4; *DcINVCW4*, *DcINVCW5*, and *DcINVCW9* each lack motif 8; and *DcINVCW10* is deficient in motif 3. Interestingly, motif 4 in *DcINVCW8* appears twice within the sequence, in contrast to the other motifs, which are present only once. Furthermore, an analysis of the intron–exon structure shows that *DcINVCW3* and *DcINVCW10* each contain five exons, whereas *DcINVCW2* and *DcINVCW6* have eight and six exons, respectively; the remaining DcINVCWs all consist of seven exons. Both *DcINVVR1* and *DcINVVR2* contain seven exons, while *DcINVVR3* has eight exons. All DcINVVRs encompass motifs 1–10. Additional analysis, which integrates gene structure with the locations of conserved motifs, indicates that motifs 1, 3, 4, 5, 6, 8, and 9 belonged to the Glyco-hydro-32N domain, whereas motifs 2, 7, and 10 belonged to the Glyco-hydro-32C domain (Figure 2C).

A total of six *INVAN* genes were identified in carrots. All *DcINVAN* genes contain the Glyco-hydro-100 domain (Figure 2C). Analysis of conserved motifs revealed that each DcINVAN protein comprises motifs 1–10, all of which are situated within the Glyco-hydro-100 domain (Figure 2C). Notably, α-family invertases possess a longer amino acid sequence upstream of the first motif (Motif 9), referred to as the N-terminal extension region; this sequence imparts unique regulatory mechanisms or functional characteristics to α-family members. Furthermore, analysis of exon structure indicates that *DcINVAN2*, *DcINVAN4*, and *DcINVAN5*, which encode α-family invertases, contain 6–7 exons, whereas *DcINVAN1*, *DcINVAN3*, and *DcINVAN6*, which encode β-family invertases, contain only 5 exons.

### 3.4. Chromosomal Distribution and Collinearity Analysis of SPS, SUS, and INV Genes

Chromosomal mapping analysis of the *DcSPS* family genes revealed that *DcSPS1*, *DcSPS2*, *DcSPS3*, *DcSPS4*, and *DcSPS5* are located on chromosomes 3, 4, 5, 6, and 7, respectively (Figure 3A). *DcSPS2*-*AtSPS4F*, *DcSPS3*-*AtSPS1F*, and *DcSPS1*-*OsSPS6* in carrot, Arabidopsis, and rice, respectively, were identified as colocalized gene pairs (Figure 3B).

The eight SUS genes in carrot are distributed on chromosomes 1, 3, 4, 7, and 8. Among them, *DcSUS2*, *DcSUS4*, and *DcSUS5* are located on chromosome 1, whereas *DcSUS1* and *DcSUS7* are located on chromosome 7 (Figure 4A). Synteny analysis among carrot, Arabidopsis, and rice identified seven syntenic gene pairs: *DcSUS2*–*AtSUS2*, *DcSUS2*–*AtSUS3*, *DcSUS4*–*AtSUS2*, *DcSUS6*–*AtSUS5*, *DcSUS7*–*AtSUS1*, *DcSUS7*–*AtSUS4*, and *DcSUS7*–*OsSUS3* (Figure 4B).

A total of 19 *INV* genes, comprising 13 *AINVs* and 6 *NINVs*, were identified in carrot. These genes were unevenly distributed across seven chromosomes, with 4, 6, 3, and 2 genes on chromosomes 1, 2, 4, 5, and 6, respectively, and only one INV gene on each of chromosomes 3 and 7 (Figure 5A). Synteny analysis revealed 17 syntenic gene pairs between carrot and Arabidopsis thaliana: *DcINVCW1*–*AtCWINV6*, *DcINVCW2*–*AtVI1*, *DcINVCW4*–*AtCWINV2*, *DcINVCW4*–*AtCWINV4*, *DcINVCW6*–*AtCWINV2*, *DcINVCW6*–*AtCWINV4*, *DcINVCW9*–*AtCWINV2*, *DcINVCW9*–*AtCWINV4*, *DcINVVR1*–*AtVI1*, *DcINVVR1*–*AtATVI2*, *DcINVVR2*–*AtVI1*, *DcINVVR2*–*AtATVI2*, *DcINVAN1*–*AtAN-INVF*, *DcINVAN1*–*AtAN-INVG*, *DcINVAN2*–*AtAN-INVC*, *DcINVAN3*–*AtAN-INVB*, and *DcINVAN5*–*AtAN-INVE*. Additionally, four syntenic gene pairs were identified between carrot and rice: *DcINVCW4*–*OsCIN3*, *DcINVCW6*–*OsCIN3*, *DcINVCW9*–*OsCIN3*, and *DcINVAN5*–*OsNIN4* (Figure 5B).

### 3.5. Duplication Events and Ka/Ks Analysis of the SPS, SUS, and INV Genes

Gene duplication plays a crucial role in the expansion of gene families and subsequent evolutionary processes in plants, with segmental and tandem duplications serving as the primary mechanisms [49]. The Ka/Ks ratio is a significant indicator for evaluating selective pressure on protein sequences throughout gene evolution, where a Ka/Ks ratio greater than 1 indicates positive selection, a ratio equal to 1 indicates neutral selection, and a ratio less than 1 indicates purifying selection [50]. Analysis of gene duplication revealed two pairs of segmentally duplicated genes (*DcSPS1*/*DcSPS3* and *DcSPS1*/*DcSPS4*) within the carrot SPS family, with Ka/Ks values of 0.11 and 0.16, respectively—both values being less than 1—suggesting that the *DcSPS* genes have experienced purifying selection during evolution. Within the carrot SUS family, one segmentally duplicated pair (*DcSUS2*/*DcSUS4*) and two tandemly duplicated pairs (*DcSUS1*/*DcSUS7* and *DcSUS2*/*DcSUS5*) were identified. The Ka/Ks value for the segmentally duplicated gene pair was 0.13, while those for the tandemly duplicated gene pairs were 0.10 and 0.11, respectively—all being less than 1—indicating that members of the *DcSUS* family have similarly undergone purifying selection during evolution. Among the members of the *DcINV* family, 10 segmentally duplicated gene pairs (*DcINVCW2*/*DcINVCW3*, *DcINVCW2*/*DcINVVR2*, *DcINVCW3*/*DcINVVR2*, *DcINVCW4*/*DcINVCW6*, *DcINVCW4*/*DcINVCW9*, *DcINVCW5*/*DcINVCW8*, *DcINVCW7*/*DcINVCW6*, *DcINVCW6*/*DcINVCW9*, *DcINVAN2*/*DcINVVR2*, and *DcINVAN3*/*DcINVAN6*) and 2 tandemly duplicated gene pairs (*DcINVVR1*/*DcINVCW3* and *DcINVVR3*/*DcINVCW8*) were identified. The Ka/Ks ratios for the segmentally duplicated gene pairs ranged from 0.11 to 0.29 (Figure 6, Table S1), while those for the tandemly duplicated gene pairs were 0.27 and 0.17, respectively, all being less than 1. This observation suggests that the *DcINV* genes have undergone purifying selection during evolution.

### 3.6. Promoter Cis-Acting Element Analysis of DcSPS, DcSUS, and DcINV Genes

To investigate the transcriptional regulatory mechanisms of the *DcSPS*, *DcSUS*, and *DcINV* gene families, we predicted cis-acting elements in their promoter regions, identifying a total of 54 cis-elements. In addition to the core promoter elements required for transcription initiation, namely the TATA-box and CAAT-box, the remaining 52 elements were categorized into four distinct groups: light-responsive elements, elements involved in plant growth and development, hormone-responsive elements, and stress-responsive elements.

Notably, light-responsive elements were the most prevalent cis-acting elements found in the promoter regions of the *DcSPS*, *DcSUS*, and *DcINV* family members (Figure 7, Table S2), encompassing Box4, G-box, and various other light-responsive motifs. Additionally, a variety of hormone-responsive elements were detected in the promoters of these genes, including abscisic acid-responsive elements (ABREs), salicylic acid-responsive elements (TCA-element), methyl jasmonate-responsive elements (TGACG-motif and CGTCA-motif), and gibberellin-responsive elements (TATC-box and P-box). Among these, ABRE exhibited the widest distribution, with genes such as *DcSPS2*, *DcSUS3*, *DcINVCW10*, *DcINVVR3*, *DcINVAN2*, and *DcINVAN5* each containing 5–7 ABRE motifs. Furthermore, stress-responsive elements, including LTR, ARE, and MBS—which are associated with responses to low temperature, oxidative stress, and drought—were also identified. With the exception of *DcSPS1* and *DcINVAN6*, the promoters of all other genes contained all four types of cis-acting elements, suggesting that the expression of the carrot *DcSPS*, *DcSUS*, and *DcINV* genes is co-regulated by multiple signaling pathways.

### 3.7. Analysis of Tissue-Specific Expression of Carrot SPS, SUS, and INV Genes

To obtain a descriptive overview of the organ-level (bulk) transcript abundance of the *DcSPS*, *DcSUS*, and *DcINV* gene families, we retrieved RNA-seq data from 20 carrot tissue samples available in the NCBI database [38] and analyzed their temporal and spatial expression profiles. Within the *SPS* family, *DcSPS1* exhibited higher expression levels in shoots, callus, and stressed roots, while *DcSPS2* showed elevated expression in flowers and leaves (Figure 8A, Tables S3 and S4). In the *SUS* family, *DcSUS1*, *DcSUS2,* and *DcSUS8* demonstrated higher overall expression levels compared to other members, with their transcripts predominantly localized to the root phloem, root xylem, fibrous roots, and leaves at the reversible wilting point (Figure 8B, Tables S3 and S4). For the INV family, *DcINVCW6*, *DcINVVR2*, *DcINVVR3*, and *DcINVAN1* were highly expressed in most examined tissues, including leaves and roots. Notably, *DcINVCW6* exhibited predominant expression in fibrous roots, whereas *DcINVVR2* showed higher transcript abundance in non-stressed leaf samples (Lf1–Lf3) and in root samples annotated as ‘stressed roots’ (Rts) in the source dataset than other family members (Figure 8C; Tables S3 and S4). Additionally, *DcINVAN1* and *DcINVAN2* were broadly expressed across various tissues.

To characterize the spatiotemporal expression dynamics of the *DcSPS*, *DcSUS*, and *DcINV* gene families throughout carrot development, we profiled transcript levels of selected family genes. The RT-PCR results were largely consistent with the expression trends identified through RNA-seq data (Figure 9). Within the *SPS* family, the transcript levels of *DcSPS1* in roots increased gradually throughout plant development, peaking at 120 days, and showed higher relative transcript abundance in roots compared to leaves at all stages. *DcSPS2* displayed significantly elevated expression in both leaves and roots at 80 days relative to other stages, while *DcSPS5* consistently showed greater expression in roots than in leaves. In the *SUS* family, *DcSUS1*, *DcSUS2*, and *DcSUS3* demonstrated higher overall expression in leaves compared to roots, with *DcSUS1* and *DcSUS2* reaching their peak transcript levels in leaves at 100 days after sowing, corresponding to the rapid taproot enlargement stage. The expression of *DcSUS3* remained relatively stable across all stages. Within the *INV* family, *DcINVVR3* was predominantly expressed in roots, whereas the expression levels of other members were generally comparable between roots and leaves. In leaves, transcript levels gradually declined over time, while in roots, they remained relatively stable, with some members exhibiting a marked increase during specific developmental stages.

### 3.8. Analysis of Expression Responses to Environmental and Hormonal Signals

To investigate the expression responses of carrot *SPS*, *SUS*, and *INV* family members to salt stress, we analyzed their transcript levels by RT-qPCR. The results show that *DcSPS* genes generally exhibited a transient increase in transcript abundance followed by a decrease in roots, whereas their expression in leaves remained relatively stable (Figure 10A–C). The *DcSUS* family genes demonstrated relatively weak responses to salt stress (Figure 10D–F). In contrast, the *DcINV* family members exhibited distinct, subfamily-specific expression patterns. The expression patterns of the *DcINVVR* and *DcINVCW* subfamily members were broadly similar in both leaves and roots, suggesting that they may fulfill comparable functions in these tissues. *DcINVAN1* displayed higher expression in the roots than in the leaves. Both *DcINVAN2* and *DcINVAN5* were significantly upregulated in the roots at 6 h, while their expression in the leaves exhibited opposite trends at the same time point: salt stress significantly suppressed *DcINVAN2* expression while promoting *DcINVAN5* expression (Figure 10G–N).

ABA acts as a core signaling phytohormone orchestrating plant stress responses. To dissect how exogenous ABA modulates sugar metabolic genes in carrot, we treated plants with 50 µM ABA and quantified transcript levels of representative *DcSPS*, *DcSUS* and *DcINV* family members via RT-PCR. The results indicated that, in leaves, the expression of *DcSPS* family genes was generally downregulated. In contrast, *DcSUS* family genes exhibited a generally weaker response to ABA treatment. Notably, all *DcINV* family genes showed a pronounced response to ABA in roots: *DcINVVR1*, *DcINVVR2*, *DcINVAN2*, and *DcINVAN5* were significantly upregulated at 6 h post-treatment, whereas *DcINVVR3*, *DcINVCW7*, and *DcINVAN1* displayed increased expression at 24 h post-treatment. Conversely, in leaves, the expression of *DcINVVR1*, *DcINVVR3*, and *DcINVCW7* was downregulated.

## 4. Discussion

SPS, SUS, and INV are essential enzymes that regulate sugar metabolism in plants, and their gene families have been identified in Arabidopsis [12], rice [22], and tomato [11]. Carrots rank among the most widely cultivated root vegetables globally, with production steadily increasing over the past five decades (data from FAO). The roots of carrots are abundant in soluble sugars, as well as β-carotene, dietary fiber, potassium, and various vitamins [51]. In this study, we systematically identified the *SPS*, *SUS*, and *INV* gene families involved in carrot sugar metabolism, analyzing their structural characteristics and evolutionary relationships. Additionally, we investigated the expression profiles of these genes across different developmental stages, as well as under salt stress and ABA treatment. These findings provide a molecular basis for elucidating the regulatory mechanisms of sugar metabolism in carrots and may facilitate future genetic improvements in root quality.

### 4.1. Structure and Evolution of the DcSPS Gene

The plant *SPS* gene family is generally small, with most species containing three to five members [12]. SPS catalyzes the condensation of UDP-glucose and fructose-6-phosphate to produce sucrose-6-phosphate, serving as the rate-limiting enzyme in the sucrose biosynthesis pathway [52]. By regulating the efficiency of photosynthetic carbon allocation to sucrose, SPS activity directly influences source–sink relationships, osmotic regulation, and the accumulation of soluble sugars in fleshy roots [53]. In this study, five *DcSPS* genes were identified in carrot (Table 1). Phylogenetic analysis revealed that these genes fall into three subfamilies (A, B, and C), with no members of subfamily D detected (Figure 1A). Previous studies have indicated that subfamilies A, B, and C are widely distributed among monocots and dicots, while subfamily D is restricted to certain monocots, such as rice [15]. The absence of subfamily D in carrot aligns with this observation. Furthermore, the presence of both subfamilies B and C in representative monocot and dicot species, including rice, Arabidopsis, and carrot, suggests that the diversification of the SPS gene family may have occurred prior to the divergence of monocots and dicots.

In carrots, all 5 *DcSPS* members contain the Glyco_transf_1 and S6PP domains, which is consistent with the typical domain architecture of the SPS family [15]. Within the *DcSPS* gene family, *DcSPS1*, *DcSPS3*, and *DcSPS4* exhibit identical exon–intron organization (Table 1; Figure 2A), suggesting that these members may have originated from an ancestral duplication event. In contrast, *DcSPS2* and *DcSPS5* show significant differences in their exon–intron structures: *DcSPS2* contains an additional exon corresponding to the fourth intron, while *DcSPS5* lacks the second exon. Intron gain and loss can be driven by various mechanisms, including transposon insertion, retrotransposition, and exonization [54]. These structural variations may influence gene transcription, mRNA stability, or translational regulation [55]. Similar phenomena have also been reported in the lychee *SPS* gene family [56]. These observations indicate that variations in intron numbers may represent a relatively common structural feature of *SPS* genes during species evolution, potentially driving functional diversification and adaptive evolution among family members. Furthermore, sequence analysis has revealed that *DcSPS3* and *DcSPS4* possess longer 3′-UTRs. Previous studies have demonstrated that differences in 3′-UTR length can affect mRNA stability and translation efficiency [57]. Based on the structural variations described above, we hypothesize that *DcSPS2* and *DcSPS5* may fulfill specialized roles in sucrose phosphate synthase activity during specific developmental stages or under environmental stress; however, this hypothesis requires further functional validation.

Gene duplication is a fundamental mechanism driving biological evolution, with both tandem and segmental duplications having profound impacts on the size and genomic distribution of gene families [49]. This study identified two segmentally duplicated gene pairs: *DcSPS1*/*DcSPS3* and *DcSPS1*/*DcSPS4*. The Ka/Ks ratios for both pairs were less than 1, indicating that these genes have undergone purifying selection [50], which is consistent with previous findings in apple [58]. These duplicated genes may play similar and functionally redundant roles in sucrose metabolism. Furthermore, interspecies collinearity analysis identified three collinear gene pairs among carrot, Arabidopsis, and rice: *DcSPS2*-*AtSPS4F*, *DcSPS3*-*AtSPS1F*, and *DcSPS1*-*OsSPS6* (Figure 3B). The presence of these syntenic relationships suggests that the corresponding SPS genes predate the divergence of monocots and dicots, thereby highlighting their evolutionary conservation across species.

### 4.2. Structure and Evolution of the DcSUS Gene

SUSs catalyze the reversible cleavage of sucrose into UDP-glucose and fructose and directly regulate carbon allocation and energy balance between source and sink organs [16]. The size of the *SUS* gene family varies significantly among species; for instance, Arabidopsis thaliana has six members [16], while pear possesses as many as 30 [59]. In this study, we identified eight *DcSUS* genes in carrots, and phylogenetic analysis classified them into three subfamilies: *SUSI*, *SUSII*, and *SUSIII* (Figure 1B). Cross-species comparisons revealed that all three subfamilies are represented in both monocots (e.g., rice) and dicots (e.g., Arabidopsis and carrot) (Figure 1B), indicating that the diversification of the SUS subfamilies predates the divergence of monocots and dicots. All members of the DcSUS family contain a Sucrose_synth domain at the N-terminus and a Glyco_transf_1 domain at the C-terminus. The N-terminal regulatory region features a conserved phosphorylation site, which modulates the enzyme’s catalytic activity and its binding to membrane systems through phosphorylation/dephosphorylation modifications [60]. The Glyco_transf_1 domain, belonging to the glycosyltransferase GT-B superfamily, is responsible for the reversible catalytic activity of SUS [47]. The exon–intron structure of the carrot SUS family is generally conserved, with each member of the *SUSI* subfamily containing 14 exons and 13 introns (Table 1, Figure 2B). Within the *SUSII* subfamily, *DcSUS4* contains an additional exon in the region corresponding to the first intron, and its 5′-UTR is significantly elongated. Exon insertions may alter the protein’s substrate-binding domains or allosteric regulation sites. Similarly, the pyruvate kinase isoenzymes PKM1 and PKM2 exhibit distinct allosteric regulatory properties through alternative splicing of exons 9 and 10 [61], suggesting that variations in exon structure represent an important mechanism for regulating enzyme function. Longer 5′-UTRs typically contain upstream open reading frames (uORFs) or translation-inhibitory elements [62], which may allow fine-tuning of *DcSUS4* translation efficiency in response to environmental signals and developmental stages. Within the *SUSIII* subfamily, *DcSUS6* lacks the first intron, resulting in a correspondingly longer first exon. Future studies could validate the effects of these structural variations on the catalytic efficiency, substrate preference, and tissue specificity of SUS through subcellular localization, enzyme kinetics, and temporal expression analysis during root development. These studies will provide potential molecular targets for elucidating the mechanisms of sugar accumulation in carrot fleshy roots.

Gene duplication serves as the primary mechanism for gene family expansion in plants. In this study, the *DcSUS* family expanded through both segmental and tandem duplications, comprising one pair of segmentally duplicated genes (*DcSUS2*/*DcSUS4*) and two pairs of tandemly duplicated genes (*DcSUS1*/*DcSUS7* and *DcSUS2*/*DcSUS5*). Notably, the Ka/Ks ratios for these duplicate gene pairs were all significantly less than 1, indicating that they have experienced strong purifying selection throughout evolution (Figure 6, Table S1). This observation aligns with findings from other plants [63]. Furthermore, six collinear gene pairs were identified between carrot and Arabidopsis, in contrast to only one pair identified with rice. This pattern is consistent with the phylogenetic divergence between dicots and monocots, indicating that *SUS* genes have undergone extensive rearrangements of chromosomal collinearity following speciation.

### 4.3. Evolution, Structure, and Classification of the DcINV Gene

INVs play a crucial role in carbon partitioning, sink organ development, and signal transduction by irreversibly catalyzing the hydrolysis of sucrose into glucose and fructose [9]. The size of the *INV* gene family varies significantly among species; for instance, sugarcane contains 14 members [64], while tomato has 19 [11]. In this study, we identified 19 *DcINV* genes in carrot, including 13 *AINVs* (comprising 10 *DcINVCWs* and 3 *DcINVVRs*) and 6 *INVANs*. Notably, the *INV* families of carrots and tomatoes are of similar size, suggesting that the expansion of this family may be influenced by comparable evolutionary constraints across different eudicots, potentially adhering to a relatively conserved evolutionary pattern. It should be noted that *LOC108209529*, encoding acid-soluble invertase isozyme II, was not captured in our current gene family analysis. This locus is of particular interest because a naturally occurring recessive mutation (rs/rs) eliminates its transcript and causes a marked shift from reducing sugars to sucrose in carrot storage roots [65]. This example highlights the functional importance of individual *INV* genes and reinforces the need for comprehensive characterization of this family in carrot.

Gene duplication serves as a significant driver of the expansion of the *DcINV* family. In our analysis, we identified 10 segmental duplication events and 2 tandem duplication events. The aforementioned duplication events explain why *DcINVCW5*, *DcINVCW8*, and *DcINVVR3* cluster together on the same branch in the phylogenetic analysis and why *DcINVVR1* and *DcINVVR2* cluster together with *DcINVCW2* and *DcINVCW3*. These sequences evolved through segmental and tandem duplication events, are phylogenetically closely related, and exhibit high sequence similarity. All DcINVCW members contain the Glyco_hydro_32N and Glyco_hydro_32C domains, while DcINVVR members additionally possess the DUF3357 domain. This domain has been shown to be essential for enzymatic activity in sweet potato vacuolar invertase [58] and is generally predicted to function as a vacuolar sorting signal involved in protein folding and targeting [8]. Therefore, we hypothesize that the DUF3357 domain may confer functional specificity to the DcINVVR subfamily, potentially affecting their vacuolar localization or protein stability. *DcINVVR3* contains eight exons, one more than *DcINVVR1* and *DcINVVR2*, which both have seven exons. This suggests that the gene may have undergone intron gain or loss events during evolution, resulting in structural divergence that could correlate with the functional specialization of *DcINVVR3*. Phylogenetic analysis and gene structure comparison indicate that the *DcINVAN* family can be divided into two subfamilies, α and β, which exhibit significant differences in exon number and coding sequence length. Members of the α subfamily encode longer proteins and are predicted to localize primarily to the chloroplast (*DcNINV2*, *DcNINV4*) and the cytoplasm (*DcNINV5*), whereas members of the β subfamily are predicted to localize to the nucleus (*DcNINV3*) and the cytoplasm (*DcNINV1*, *DcNINV6*). These distinct localization patterns suggest that precise targeting is finely regulated by specific N-terminal sorting signals. The extended N-terminal regions of α-subfamily proteins resemble chloroplast transit peptides; however, dedicated prediction tools and experimental validation are needed to confirm this. Similarly, diversity in the subcellular localization of INVAN has been reported in Arabidopsis—*AtNINV1* is localized to the cytoplasm, consistent with its role in sugar signal transduction, while chloroplast-localized isoforms are suggested to participate in the immediate utilization of photosynthetic products [45]. Elucidating whether the localization differentiation of the two DcINVAN subfamilies reflects a functional division of labor in sucrose metabolism and sugar signaling will require further experimental validation.

### 4.4. Analysis of the Tissue Expression Profiles of the DcSPS, DcSUS, and DcINV Gene Families

The differential expression of genes involved in sucrose metabolism between source and sink organs is a crucial mechanism by which plants coordinate carbon allocation and the development of sink organs. In this study, we showed that the *DcSPS*, *DcSUS*, and *DcINV* family members analyzed exhibited distinct expression patterns across various carrot tissues, suggesting that these families may act together to regulate carbon metabolism and sugar signaling during taproot development.

Within the *SPS* family, *DcSPS1* is expressed in both roots and leaves, while *DcSPS2* demonstrates preferential expression in seeds, flowers, and young leaves (Figure 8A, Tables S3 and S4). Phylogenetic analysis reveals that *DcSPS2* clusters with Arabidopsis *AtSPS4F* (*AT4G10120*) on the same branch, indicating that they may be orthologs. *AtSPS4F* has been reported to be expressed in embryos [66], and its expression pattern is consistent with a potential role in embryonic development. However, functional validation through genetic approaches is required to confirm such a role. Given the high expression of *DcSPS2* in seeds, flowers, and young leaves, we hypothesize that *DcSPS1* may sustain basal sucrose synthesis in carrots, while *DcSPS2* may participate in carbon metabolism during reproductive development and early vegetative growth.

Within the *SUS* family, *DcSUS1* and *DcSUS8* exhibit high expression levels in roots, leading us to hypothesize that they facilitate carbon supply and energy metabolism during taproot development by sustaining elevated SUS activity (Figure 8B, Tables S3 and S4). This hypothesis is reinforced by findings that strawberry *FaSUS1* and tomato *TOMSSF* regulate carbon supply via SUS activity during fruit development [67,68], indicating a potential functional conservation across species. Additionally, *DcSUS2* is predominantly expressed in floral organs, which is in accordance with previous observation [69]. Phylogenetic analysis reveals that *DcSUS2* clusters with Arabidopsis *AtSUS3* and rice *OsSUS4*. In *Arabidopsis*, *AtSUS3* is expressed during late seed maturation and under water stress, suggesting its involvement in seed development [16], while *OsSUS4* in rice is highly expressed in developing pome, where it plays a role in carbon allocation during grain filling and regulates endosperm starch synthesis [17]. Given the high expression of *DcSUS2* in floral organs, we speculate that it may be involved in floral organ development. Nonetheless, its specific physiological functions require further verification through mutant phenotype analysis.

Within the *INV* family, *DcINVCW6* exhibits high transcriptional levels in the xylem and leaves, and is also highly expressed in callus tissue. Studies in maize have demonstrated that CWIN activity is essential for the normal development of endosperm and maternal tissues during seed development [70]. Consequently, we hypothesize that the elevated expression of *DcINVCW6* in carrot xylem may play a role in vascular tissue development by maintaining the apoplastic sucrose gradient, thereby facilitating phloem unloading. However, its functional role in callus tissue necessitates further validation through cytological localization and mutant analysis. *DcINVVR2* and *DcINVVR3* are consistently highly expressed in both leaves and roots (Figure 8C, Tables S3 and S4). Vacuolar invertase activity is crucial for sugar homeostasis in both source and sink organs; for instance, in tomato, the inhibitory interaction between *SlVIF* and *SlVI* regulates sucrose metabolism during fruit ripening [71]. We hypothesize that the high expression of *DcINVVR2* and *DcINVVR3* in both photosynthetic tissues (leaves) and storage tissues (roots) may regulate local sugar homeostasis through vacuolar sucrose hydrolysis and hexose accumulation; however, whether they also contribute to inter-organ sugar coordination requires further experimental verification. Furthermore, *DcINVAN1* is highly expressed across various tissues, particularly in the taproot (Figure 8C and Figure 9L). Combined with the predicted subcellular localization, the elevated expression of *DcINVAN1* in the taproot suggests that it may contribute to cytoplasmic sucrose cleavage to support metabolic demand in storage organs; however, its specific physiological function requires further elucidation. Taken together, these expression patterns suggest that the *DcSPS*, *DcSUS*, and *DcINV* families act coordinately during carrot taproot development. It should be noted, however, that the transcript abundance data reported here were obtained from bulk tissue samples, including both the RNA-seq data and the RT-qPCR analysis. Such organ-level data represent the average transcript abundance across all cell types within an organ and cannot distinguish whether a high average level arises from a larger proportion of expressing cells or from a higher expression level within individual cells. Because cell-type-resolved or single-cell data were not available for these samples, the expression patterns described here are strictly descriptive at the organ level and should not be interpreted as cell-type-specific expression. The observed organ- and stage-specific patterns of DcSPS, DcSUS, and DcINV family genes should therefore be interpreted as part of the coordinated carbon metabolism network, consistent with the integrated roles of sucrose synthesis, cleavage, and transport across organs and developmental stages. Resolving the cell-type-specific contributions to these organ-level patterns will require spatial or single-cell approaches in future studies.

### 4.5. Cis-Acting Elements in the Promoters of the DcSPS, DcSUS, and DcINV Genes, and Their Expression in Response to Treatment with Stress-Inducing Agents

Promoter cis-acting elements function as crucial regulatory switches that modulate gene transcription and play significant roles in the precise regulation of various biological processes [72]. In this study, in silico predictions of the promoter regions for the *DcSPS*, *DcSUS*, and *DcINV* gene families revealed an abundance of cis-regulatory elements across all three families. Notably, the abscisic acid response element (ABRE) was the most prevalent, indicating potential roles of these families in responding to abiotic stress in carrots. To assess the functional relevance of these promoter elements, we analyzed the expression of several *SPSs*, *SUSs* and *INVs* following NaCl and ABA treatments. It should be noted, however, that untreated control plants were sampled only at 0 h, and time-matched controls were not included at every treatment time point. Therefore, temporal or circadian effects cannot be fully excluded, and the stress-responsive expression patterns reported here should be considered putative rather than definitive. All samples were collected at the same time of day to minimize circadian variation.

Under NaCl stress, *DcSPS* genes generally exhibit a transient increase followed by a decrease in roots, whereas their expression in leaves remains relatively stable overall (Figure 10A–C). The *DcSUS* family exhibited a generally weak response to NaCl treatment (Figure 10D–F). Among the *DcINV* family, *DcINVVR2*, *DcINVAN2*, and *DcINVAN5* were upregulated during the initial stages of stress but displayed a declining trend as the treatment duration increased. These time-dependent expression fluctuations resemble the temporal response patterns of certain sucrose metabolism genes in apple leaves under drought stress [73], suggesting that the dynamic temporal responses of sucrose metabolism genes to abiotic stress may be conserved across species. Furthermore, *DcINVAN1* exhibited higher expression levels in roots compared to leaves; *DcINVAN2* and *DcINVAN5* were both significantly upregulated in roots at 6 h but displayed opposing expression trends in leaves—*DcINVAN2* was downregulated while *DcINVAN5* was upregulated under salt stress. This suggests that members of the *DcINVAN* subfamily may possess distinct regulatory expression patterns in different carrot organs. In contrast to the organ-specific responses observed in *DcINVAN* members, the salt-responsive *DcSPS* members in carrot roots displayed a transient upregulation followed by a decline (Figure 10A–C). This temporal pattern under salt stress differs from the sustained upregulation of *AtSPS2F* and *AtSPS4F* observed under osmotic stress in Arabidopsis. This discrepancy suggests that carrot may prioritize a rapid and transient enhancement of sucrose synthesis to provide immediate osmotic protection and energy during the early phase of salt stress, rather than mounting a prolonged response.

ABA treatment relies on a single concentration adopted from previous work, and a comprehensive dose–response analysis was not performed. It should be noted that solvent-matched mock controls for the ABA treatment were not included in this study. Therefore, the ABA-related results should be interpreted with caution, as a contribution from the solvent cannot be fully excluded. Under ABA treatment, members of the *DcSPS* family in leaves generally exhibited a decline in expression, while *DcSPS2* in roots was gradually upregulated over time. This pattern aligns with the upregulation of the Arabidopsis homolog *AtSPS4F* under osmotic stress [66], indicating that this response may be mediated by a conserved ABA-dependent pathway. In contrast, the *DcSUS* family did not show significant transcriptional changes under ABA treatment (Figure 11D–F). Unlike the response to NaCl stress, all detected members of the *DcINV* family responded in roots under ABA treatment; however, the timing of their response varied: *DcINVVR1*, *DcINVVR2*, *DcINVAN2*, and *DcINVAN5* were significantly upregulated at 6 h (early responders), while *DcINVVR3*, *DcINVCW7*, and *DcINVAN1* were upregulated at 24 h (late responders) (Figure 11G–N).

## 5. Conclusions

In this study, we identified 5 *DcSPS*, 8 *DcSUS*, and 19 *DcINV* genes in carrot. Phylogenetic analysis indicated that fragment duplication is the primary driver of family expansion, and that family members are subject to purifying selection. Transcriptomic and RT-PCR analyses revealed that members of the carrot *SPS*, *SUS*, and *INV* families exhibit distinct tissue-specific expression patterns. SPS and INV members showed divergent tissue-preferred expression, and SUS genes mainly expressed in leaves and roots, pointing to coordinated regulation of sucrose source–sink distribution by the three families during organ development. These families exhibited divergent stress responsiveness: root *DcSPS1/2/5* showed transient upregulation followed by repression under NaCl, *DcSPS2* was persistently induced by ABA, and SUS genes were barely responsive to either stress. Root INV members displayed time-dependent stress induction, while *DcINVVR1*, *DcINVVR3* and *DcINVCW7* were suppressed in leaves. These findings may provide valuable candidate gene resources and a theoretical reference for the molecular improvement of carrot flavor quality, stress tolerance, and precision cultivation management.

## Figures and Tables

**Figure 1:**
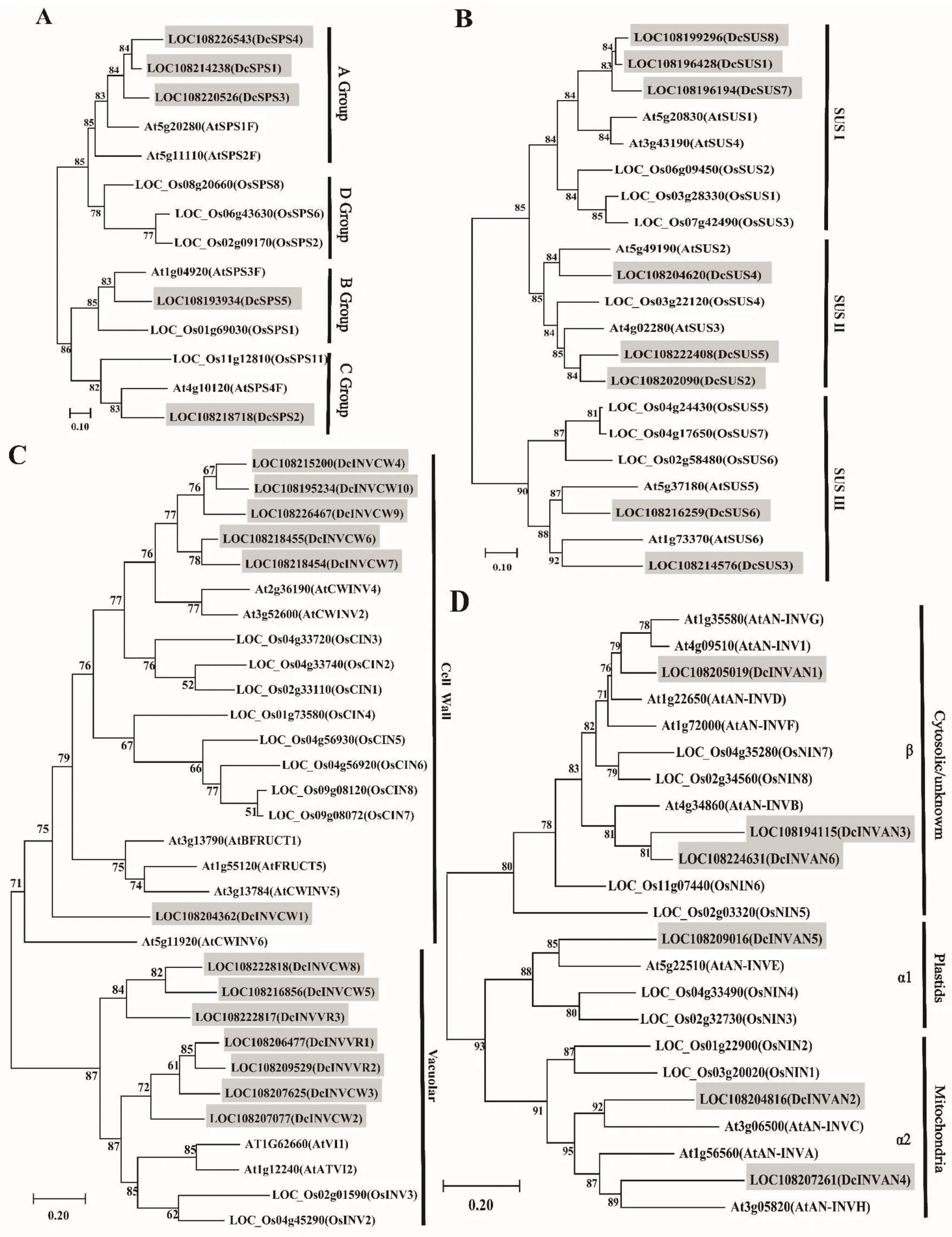
Phylogenetic trees of SPS (**A**), SUS (**B**), AINV (**C**), and NINV (**D**) constructed using the maximum likelihood method in MEGA-X, based on amino acid sequences from Arabidopsis, carrot, and rice. Carrot proteins are highlighted with a gray background.

**Figure 2:**
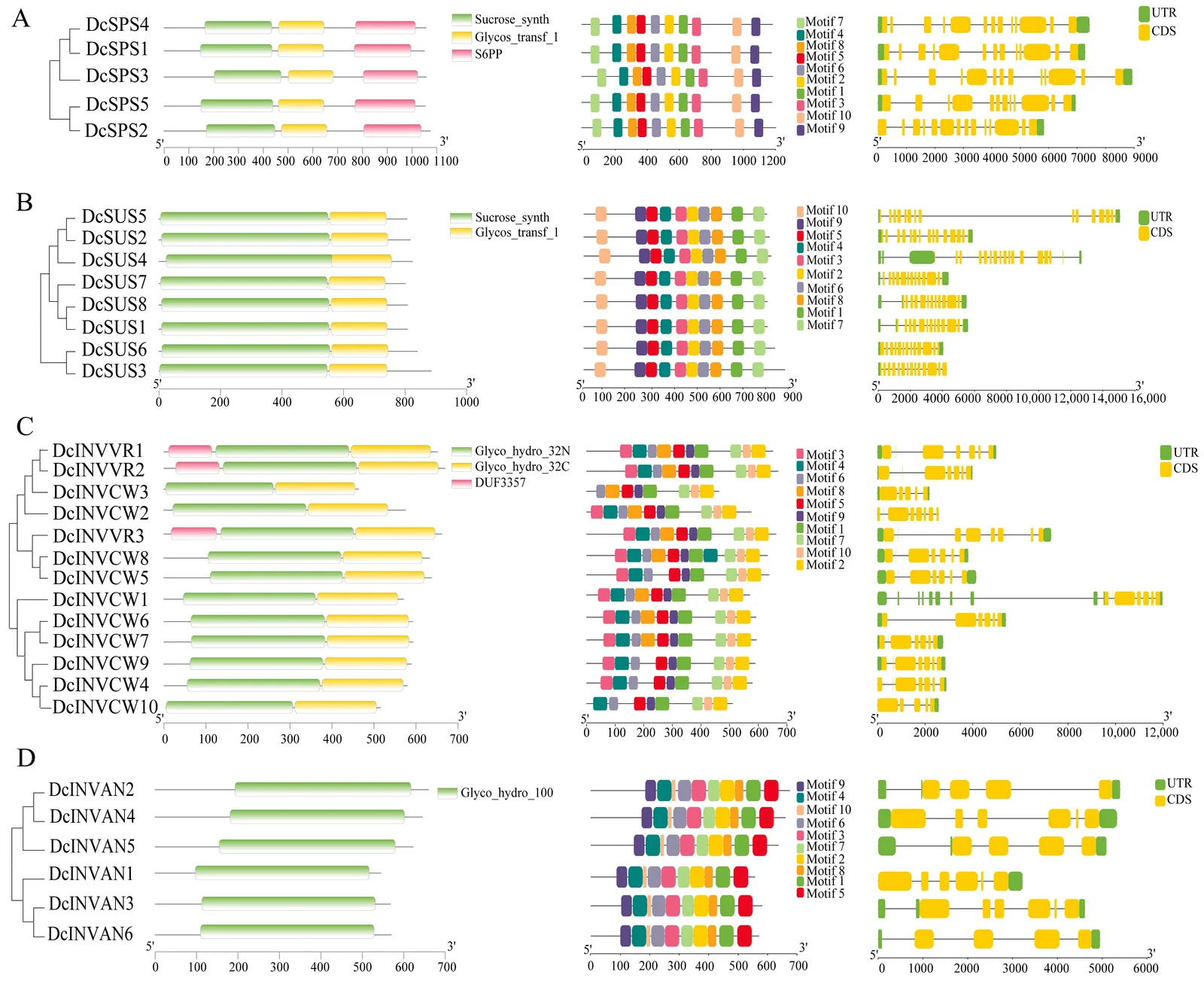
Conserved domains, conserved motifs, and gene structures of the *SPS* (**A**), *SUS* (**B**), *AINV* (**C**), and *INVAN* (**D**) families in carrot. In the conserved domain diagrams, different domains are marked with distinct colors. In the motif diagrams, different conserved motifs are represented by distinct colors. In the gene structure diagrams, green and yellow boxes represent UTRs and CDS, respectively.

**Figure 3:**
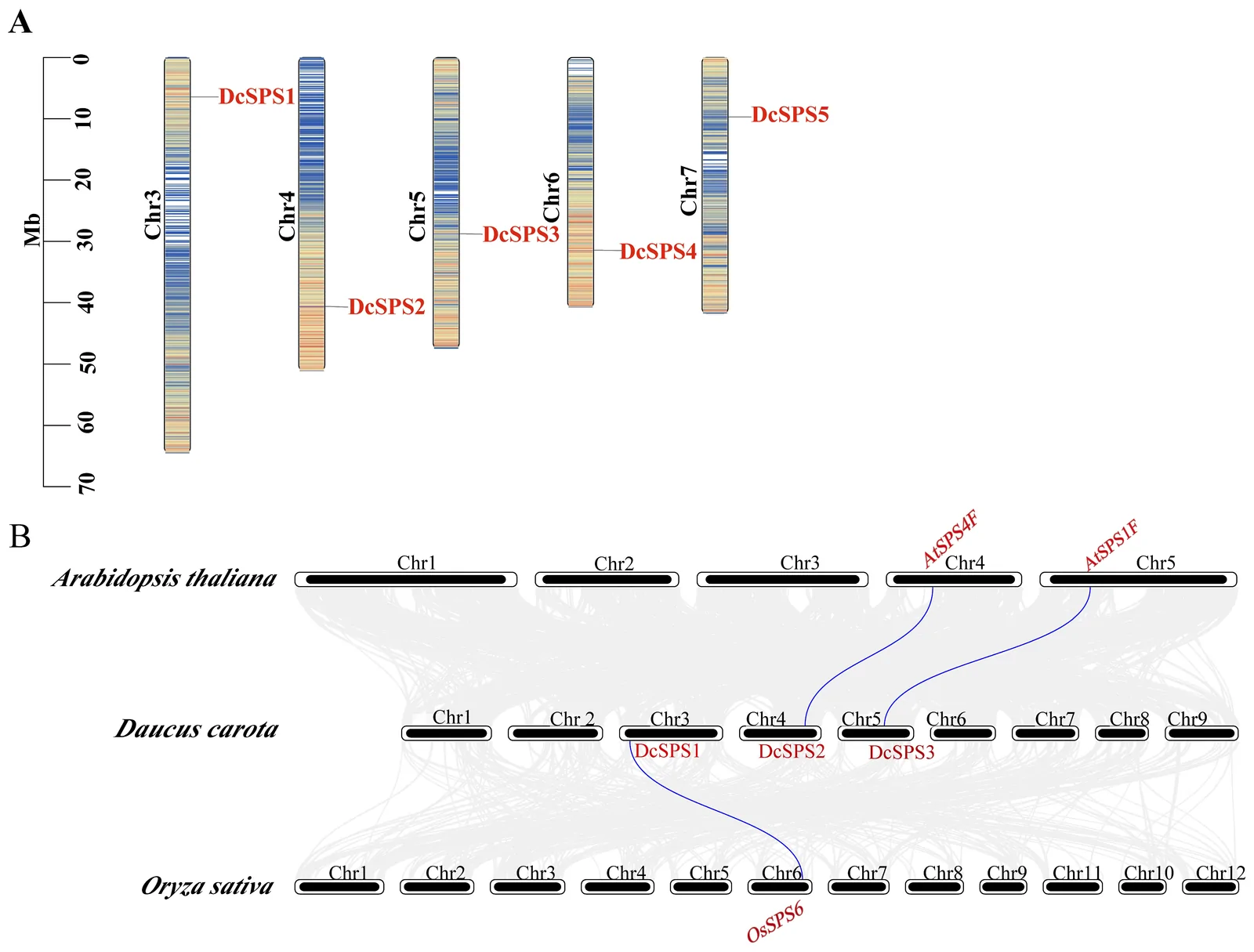
Chromosomal distribution of *SPS* genes in carrot, the vertical bars represent chromosomes, and the color stripes indicate gene density, the red lines indicate the positions of the *SPS* family members. (**A**) and syntenic relationships of *SPS* genes among carrot, Arabidopsis, and rice (**B**).

**Figure 4:**
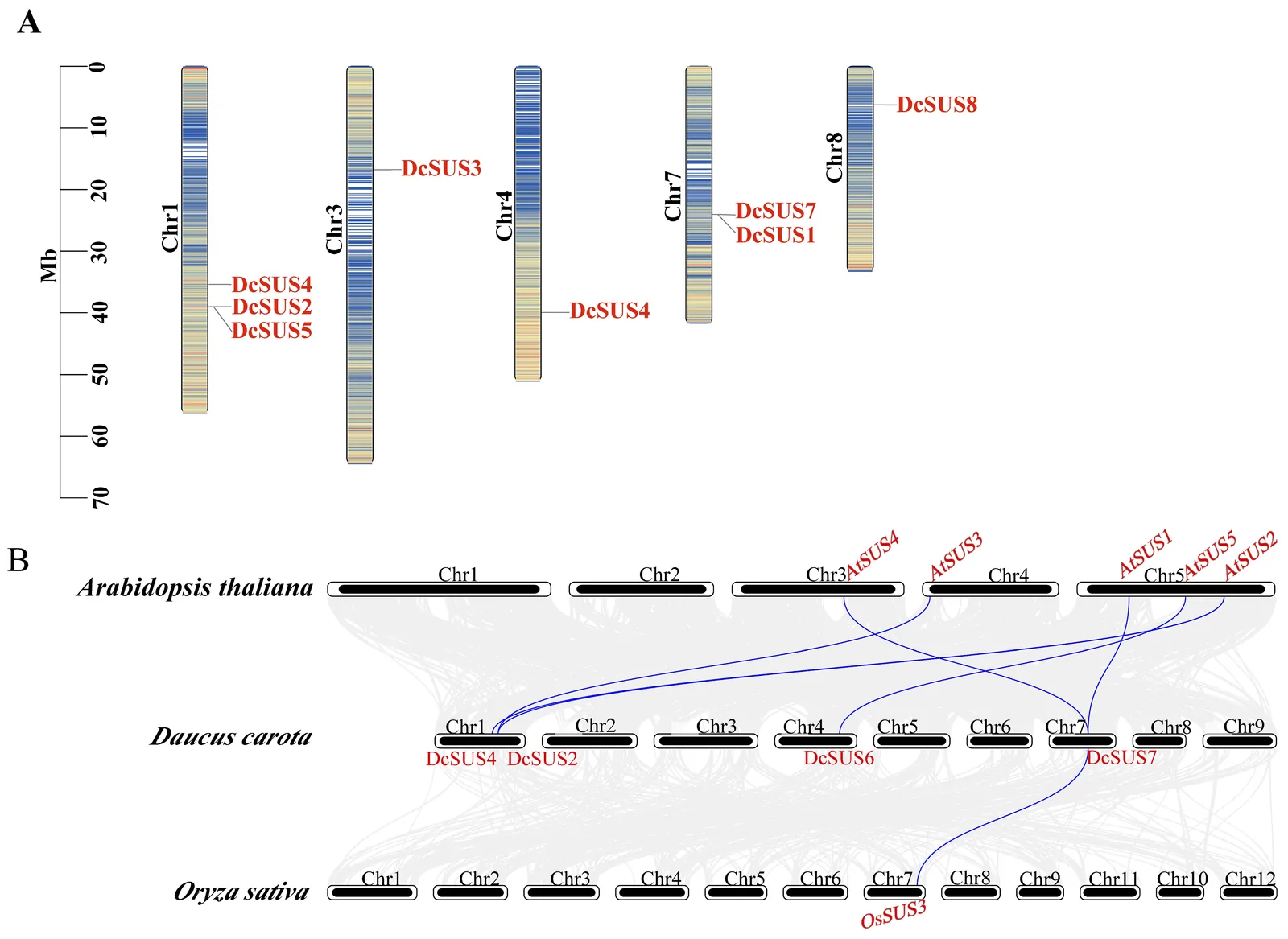
Chromosomal distribution of *SUS* genes in carrot, the vertical bars represent chromosomes, and the color stripes indicate gene density, the red lines indicate the positions of the *SUS* family members. (**A**) and syntenic relationships of *SUS* genes among carrot, Arabidopsis, and rice (**B**).

**Figure 5:**
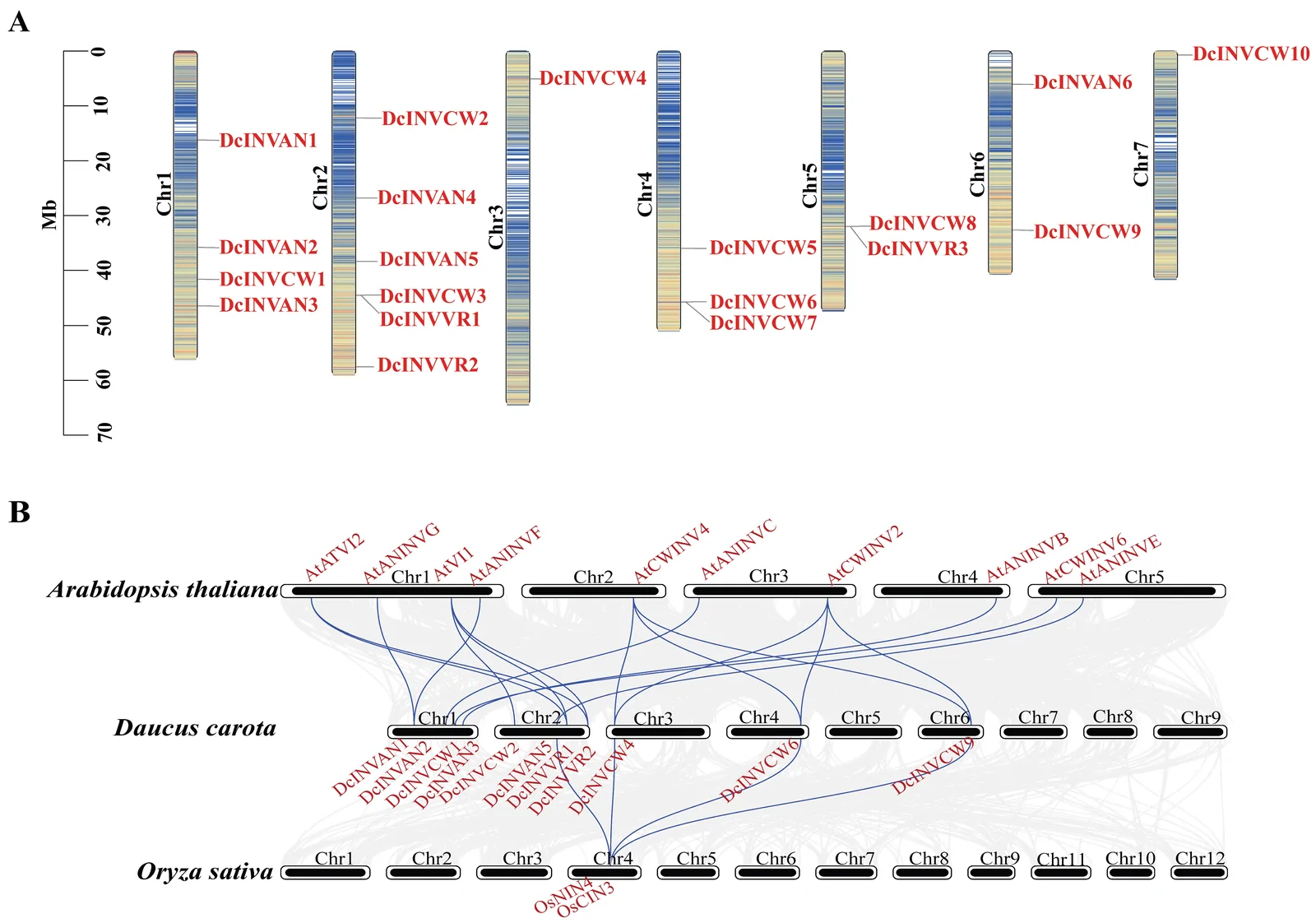
Chromosomal distribution of *INV* genes in carrot, the vertical bars represent chromosomes, and the color stripes indicate gene density, the red lines indicate the positions of the *INV* family members. (**A**) and syntenic relationships of *INV* genes among carrot, Arabidopsis, and rice (**B**).

**Figure 6:**
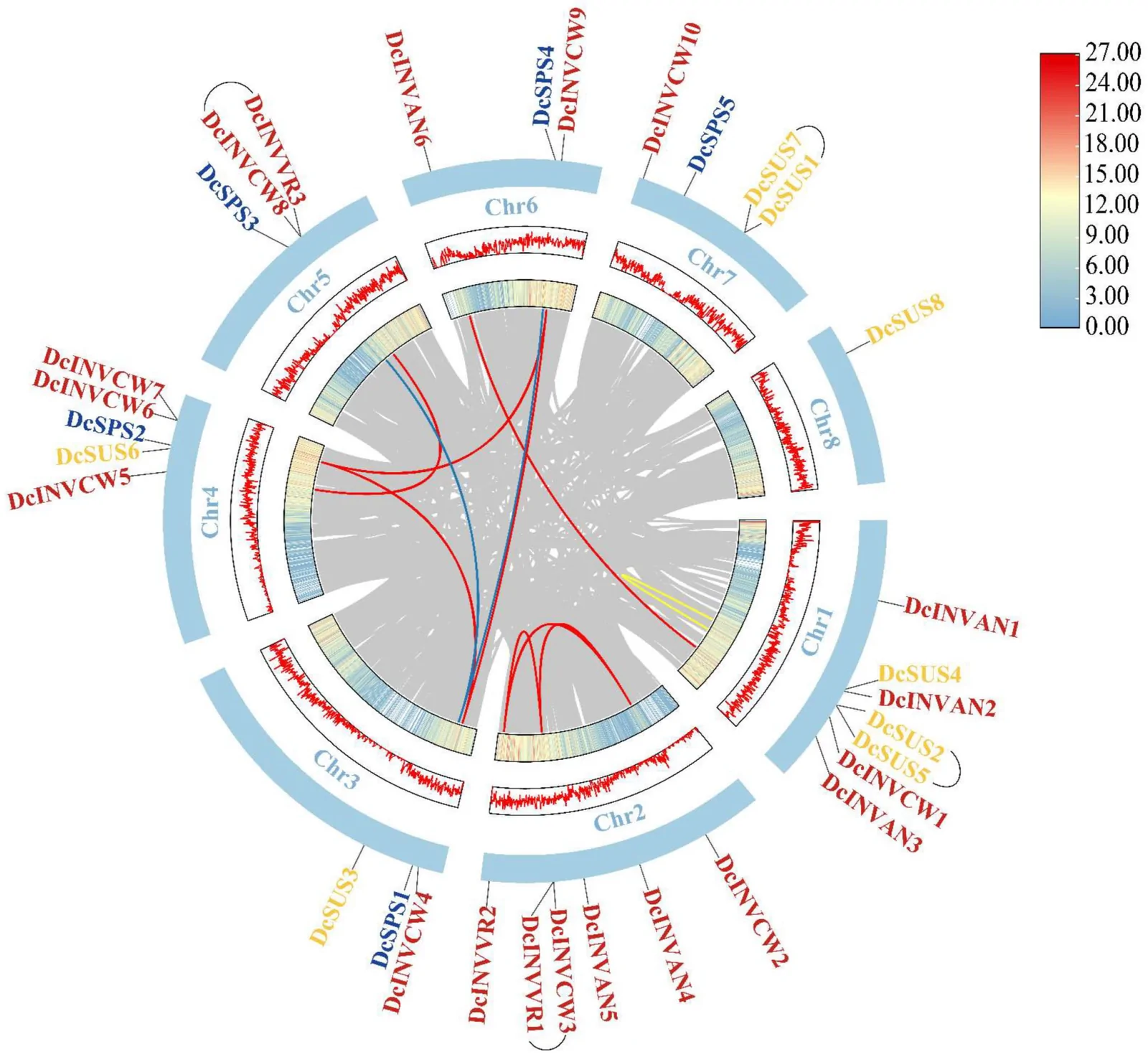
Synteny analysis of *SPS*, *SUS*, and *INV* family members in carrot. The blue bars indicate the positions of genes on the chromosomes. Different colors of gene names are used to distinguish different gene families: blue for *SPS*, yellow for *SUS*, and red for *INV*; black curved lines indicate tandemly duplicated gene pairs.

**Figure 7:**
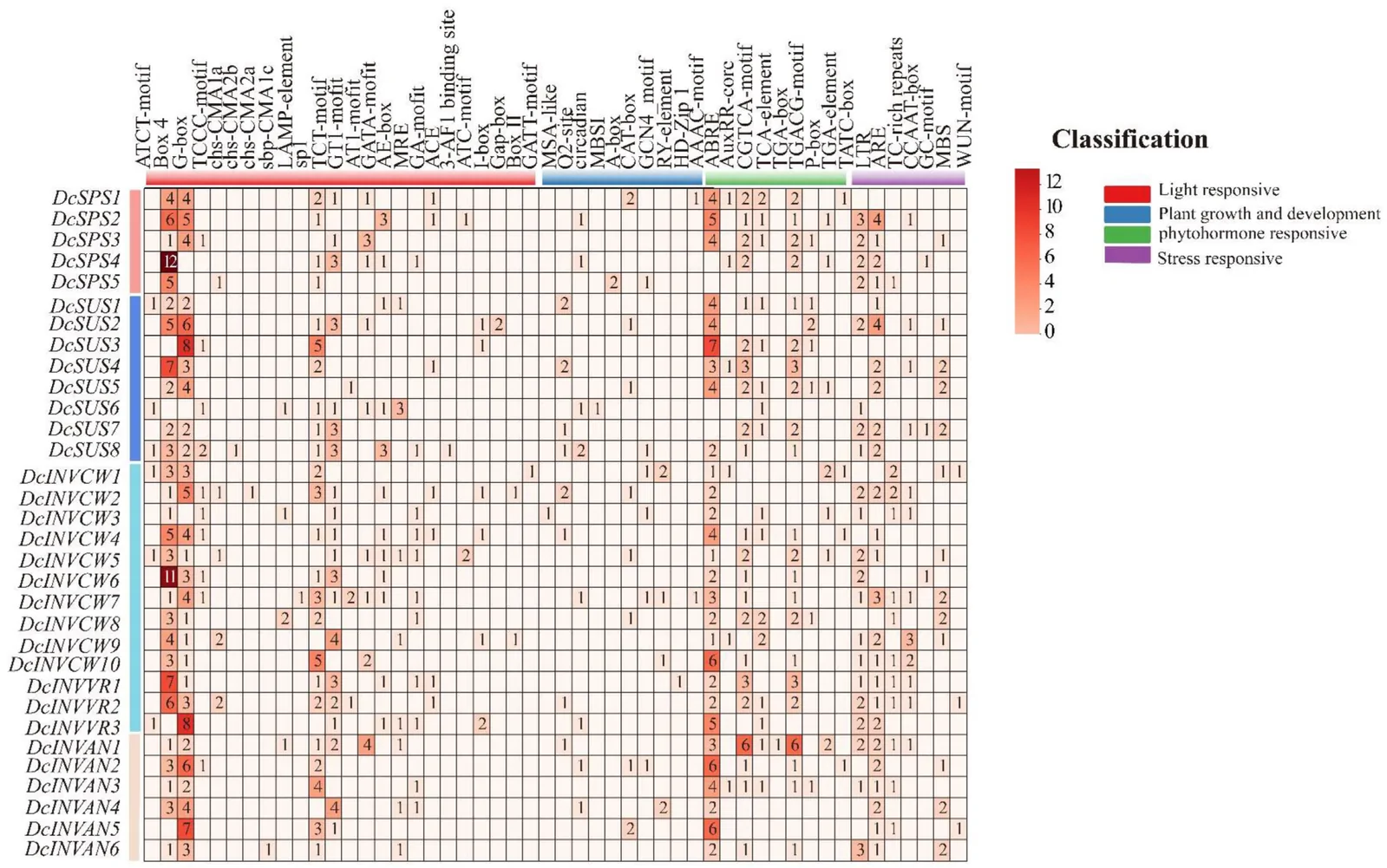
Distribution of cis-acting elements in the promoter regions of *DcSPS*, *DcSUS*, and *DcINV* genes.

**Figure 8:**
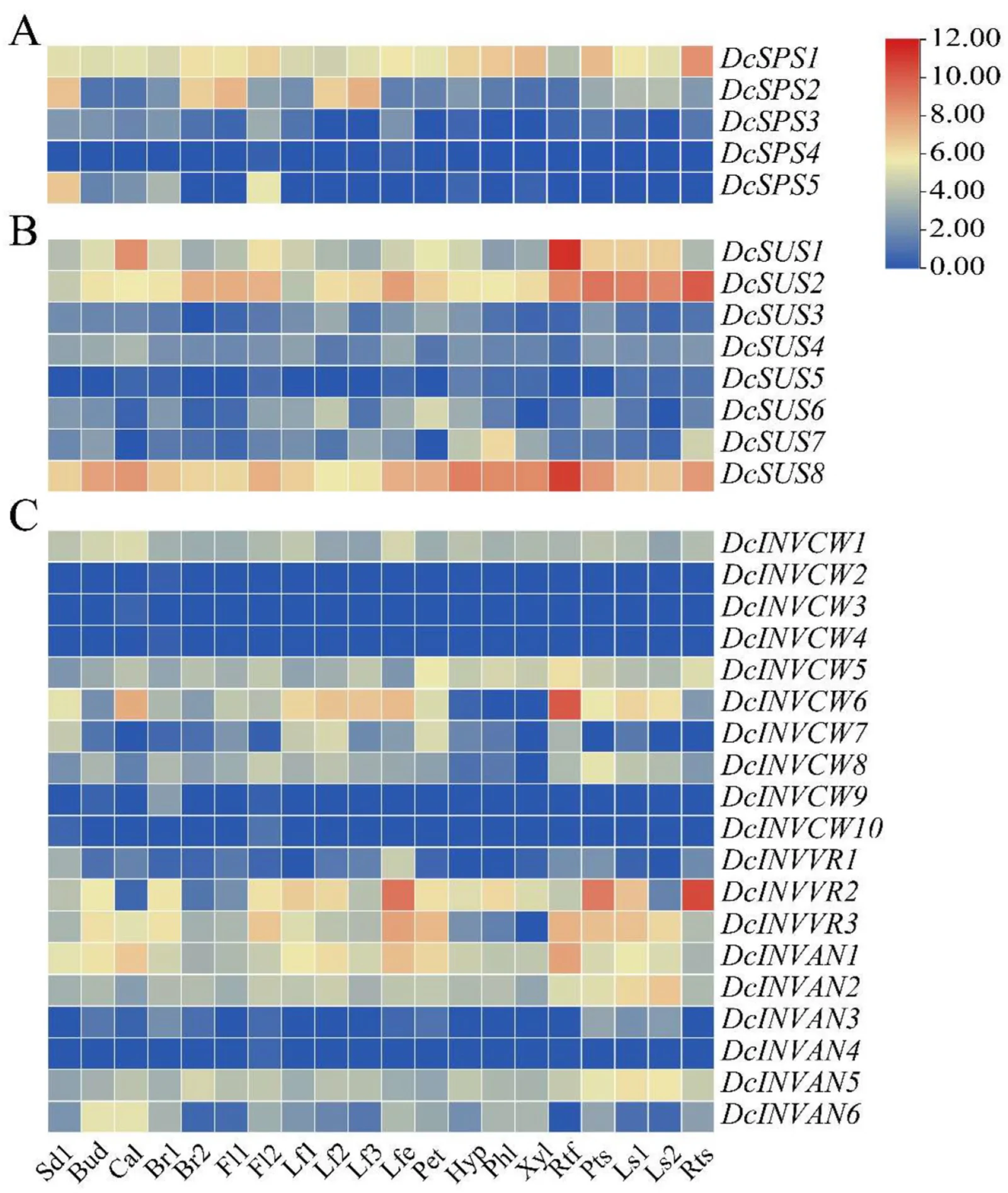
Expression profiles of (**A**) *DcSPS*, (**B**) *DcSUS*, and (**C**) *DcINV* genes in different carrot tissues. The color scale represents log-transformed TPM values. Sample abbreviations are as follows: Sd1, early germination stage seeds; Bud, 0.5 mm shoots; Cal, callus tissue; Br1 and Br2, unopened and open bracts, respectively; FI1 and FI2, unopened whole flowers and open whole flowers at peak bloom, respectively; Lf1, Lf2, Lf3, and Lfe, leaf stage 1 (0.5–1 cm plants), leaf stage 2 (2–2.5 cm leaves, not fully expanded), leaf stage 3 (7–8 cm fully expanded leaves), and yellow leaves, respectively; Pet, 10 cm petiole (from leaf stage 3); Hyp, lower hypocotyl; Phl, root phloem; Xyl, root xylem; Rtf, fibrous roots; Pts and Ls1, stressed leaves at the reversible wilting point (2–2.5 cm and 7–8 cm long, respectively); Rts, stressed roots; Ls2, stressed leaves at the reversible wilting point (2–2.5 cm and 7–8 cm long, respectively).

**Figure 9:**
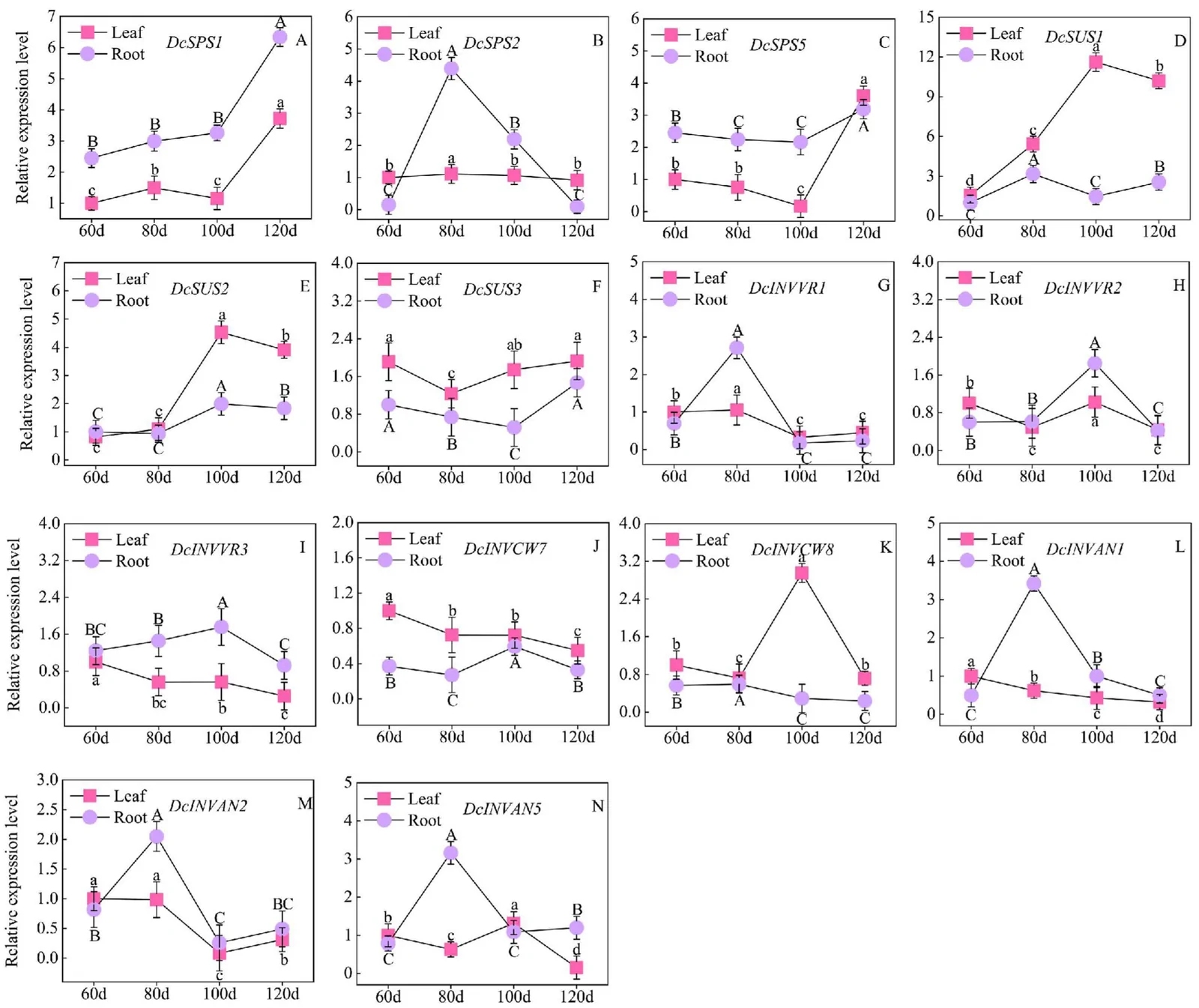
Relative mRNA abundance of *DcSPS*, *DcSUS*, and *DcINV* family genes in the roots and leaves of carrot plants at 60, 80, 100, and 120 days after sowing (DASs). Transcript levels were determined by RT-qPCR, normalized to the geometric mean of *Actin* and *5.8S rRNA*, and expressed relative to the 60-day leaf samples (calibrator) using the 2^−ΔΔCt^ method. (**A**) *DcSPS1*; (**B**) *DcSPS2*; (**C**) *DcSPS5*; (**D**) *DcSUS1*; (**E**) *DcSUS2*; (**F**) *DcSUS3*; (**G**) *DcINVVR1*; (**H**) *DcINVVR2*; (**I**) *DcINVVR3*; (**J**) *DcINVCW7*; (**K**) *DcINVCW8*; (**L**) *DcINVAN1*; (**M**) *DcINVAN2*; (**N**) *DcINVAN5*. Different lowercase letters indicate significant differences among time points in leaves, and different uppercase letters indicate significant differences among time points in roots (*p* < 0.05, one-way ANOVA with Duncan’s multiple range test). Data are presented as mean ± SD of three biological replicates.

**Figure 10:**
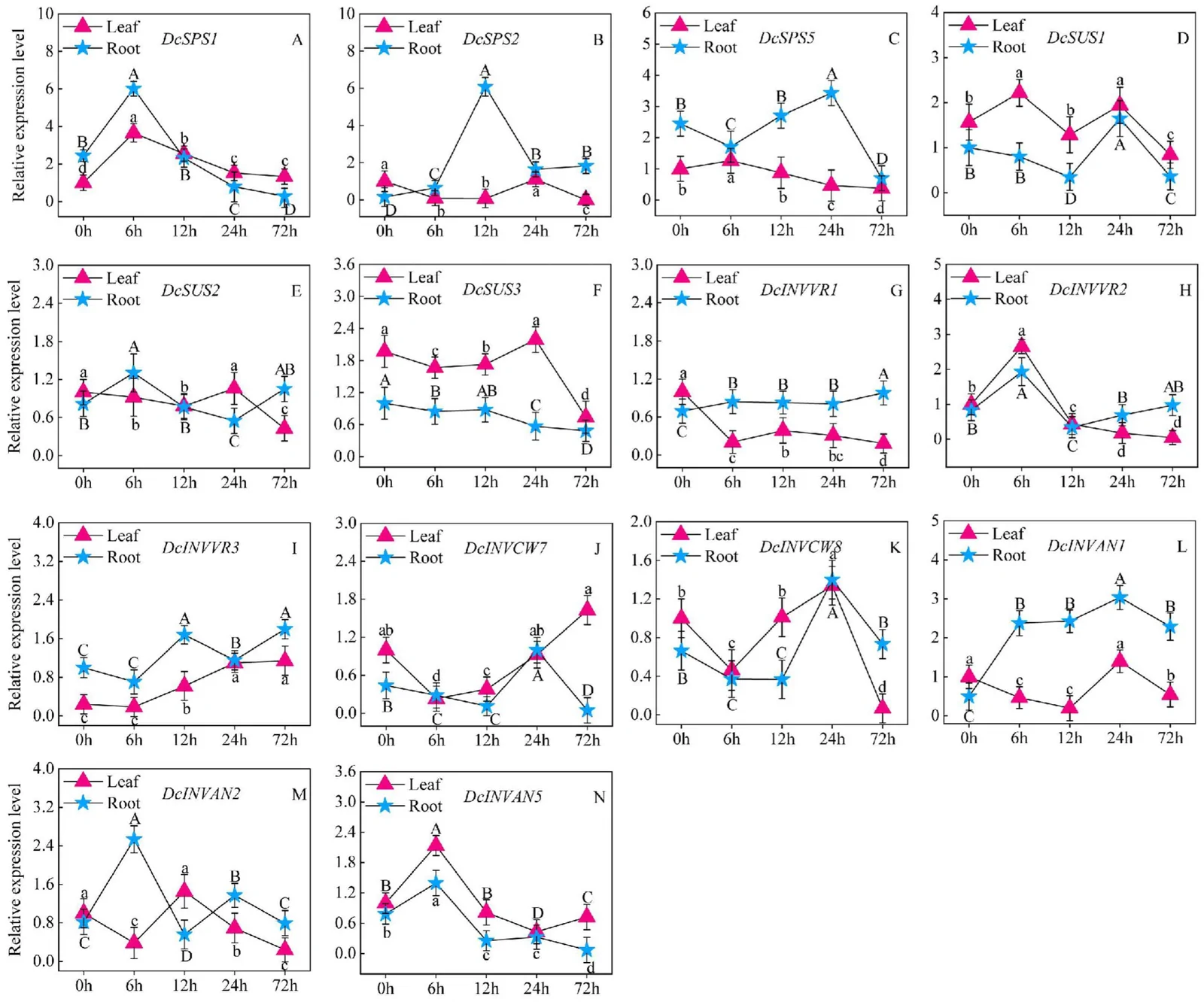
Relative mRNA abundance of *DcSPS*, *DcSUS*, and *DcINV* family genes in the roots and leaves of carrot plants in response to 150 mM NaCl stress. Transcript levels were determined by RT-qPCR, normalized to the geometric mean of *Actin* and *5.8S rRNA*, and expressed relative to the 0 h leaf sample (calibrator) using the 2^−ΔΔCt^ method. (**A**) *DcSPS1*; (**B**) *DcSPS2*; (**C**) *DcSPS5*; (**D**) *DcSUS1*; (**E**) *DcSUS2*; (**F**) *DcSUS3*; (**G**) *DcINVVR1*; (**H**) *DcINVVR2*; (**I**) *DcINVVR3*; (**J**) *DcINVCW7*; (**K**) *DcINVCW8*; (**L**) *DcINVAN1*; (**M**) *DcINVAN2*; (**N**) *DcINVAN5*. Different lowercase lettersindicate significant differences among time points in leaves, and different uppercase letters indicate significant differences among time points in roots (*p* < 0.05, one-way ANOVA with Duncan’s multiple range test). Data are presented as mean ± SD of three biological replicates.

**Figure 11:**
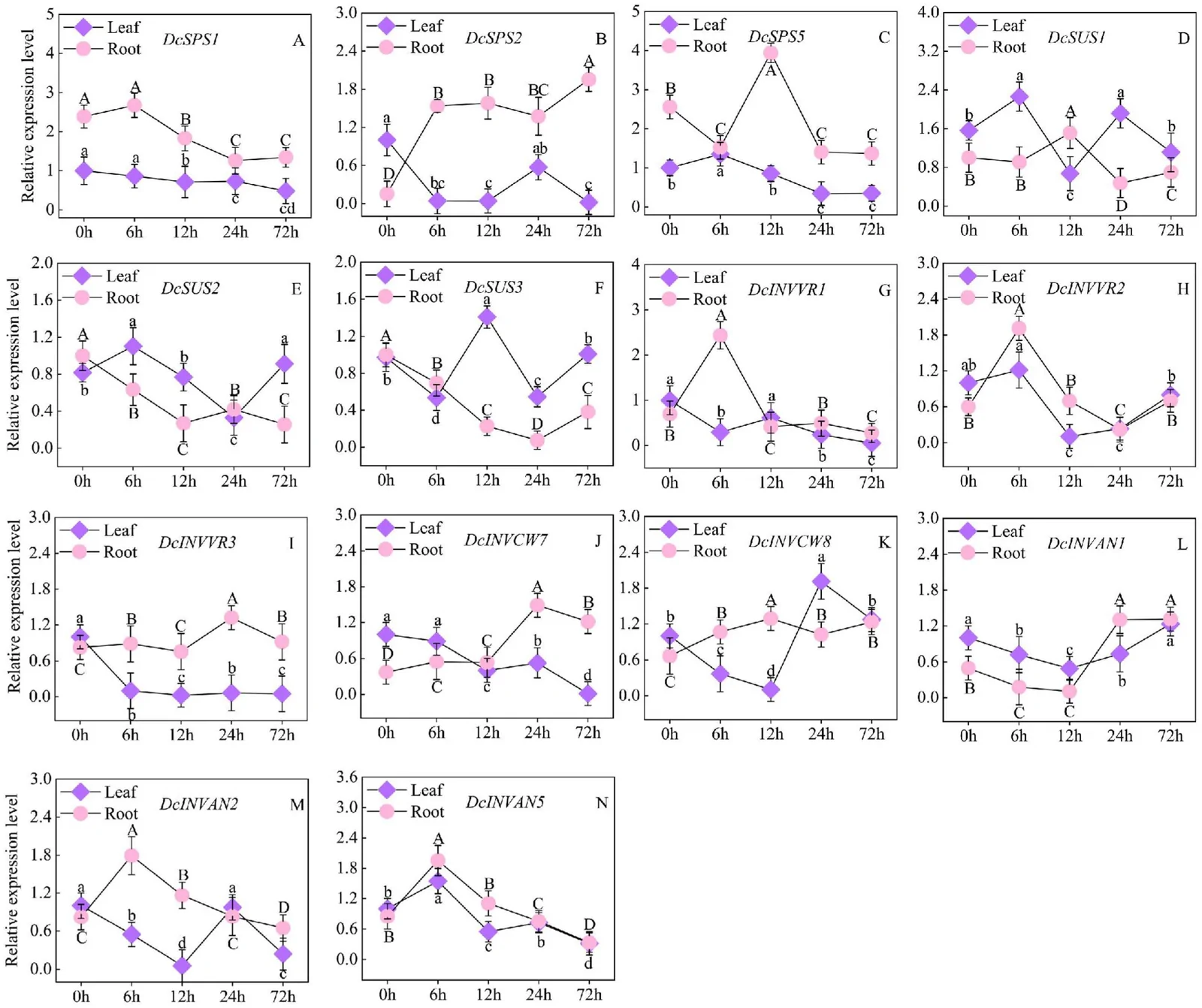
Relative mRNA abundance of *DcSPS*, *DcSUS*, and *DcINV* family genes in the roots and leaves of carrot plants in response to 50 µM ABA treatment. Transcript levels were determined by RT-qPCR, normalized to the geometric mean of *Actin* and *5.8S rRNA*, and expressed relative to the 0 h leaf sample (calibrator) using the 2^−ΔΔCt^ method. (**A**) *DcSPS1*; (**B**) *DcSPS2*; (**C**) *DcSPS5*; (**D**) *DcSUS1*; (**E**) *DcSUS2*; (**F**) *DcSUS3*; (**G**) *DcINVVR1*; (**H**) *DcINVVR2*; (**I**) *DcINVVR3*; (**J**) *DcINVCW7*; (**K**) *DcINVCW8*; (**L**) *DcINVAN1*; (**M**) *DcINVAN2*; (**N**) *DcINVAN5*. Different lowercase letters indicate significant differences among time points in leaves, and different uppercase letters indicate significant differences among time points in roots (*p* < 0.05, one-way ANOVA with Duncan’s multiple range test). Data are presented as mean ± SD of three biological replicates.

**Table 1:** Gene structure and protein characteristics of *DcSPSs*, *DcSUSs* and *DcINVs* in carrot.

Gene			Gene Structure					Protein Characteristics						Gene Name in the Literature
Name	Locus	Chr	Sequence Length (bp)	CDS Length (bp)	Exon No.	Intron No.	Length (Aa)	MW (kDa)	pI	Instability Index	Aliphatic Index	GRAVY	sl	Reference
*DcSPS1*	LOC108214238	3	7279	3162	13	12	1053	118.14	5.80	49.01	84.56	−0.441	nucl	
*DcSPS2*	LOC108218718	4	5835	3174	14	13	1057	118.98	6.01	49.02	83.86	−0.411	chlo	
*DcSPS3*	LOC108220526	5	8937	3171	13	12	1056	118.68	6.55	48.86	88.37	−0.397	nucl	
*DcSPS4*	LOC108226543	6	7431	3153	13	12	1050	118.61	5.70	45.90	89.80	−0.353	nucl	
*DcSPS5*	LOC108193934	7	6944	3225	12	11	1074	120.67	7.00	46.50	86.23	−0.470	cyto	
*DcSUS1*	LOC108196428	7	5594	2427	14	13	808	92.69	6.00	36.83	90.47	−0.259	cyto	*DcSUS1* [21]
*DcSUS2*	LOC108202090	1	5882	2454	15	14	817	93.53	5.85	38.18	90.09	−0.313	cyto	*DcSUS2* [21]
*DcSUS3*	LOC108214576	3	4271	2658	14	13	885	100.32	6.22	39.95	87.82	−0.244	nucl	*DcSUS3* [21]
*DcSUS4*	LOC108204620	1	12671	2475	16	15	824	93.98	6.03	35.43	94.76	−0.217	chlo	
*DcSUS5*	LOC108222408	1	15070	2421	15	14	806	91.86	6.30	40.03	89.99	−0.273	cyto	
*DcSUS6*	LOC108216259	4	4051	2523	13	12	840	95.20	6.94	36.85	89.64	−0.229	cyto	
*DcSUS7*	LOC108196194	7	4378	2406	14	13	801	91.65	6.33	35.90	93.96	−0.192	cyto	
*DcSUS8*	LOC108199296	8	5503	2427	14	13	808	92.47	6.21	33.31	91.45	−0.256	cyto	
*DcINVCW1*	LOC108204362	1	11973	1713	16	15	570	64.88	8.92	38.09	76.44	−0.430	chlo	
*DcINVCW2*	LOC108207077	2	2572	1728	8	7	575	64.40	7.64	27.08	78.24	−0.315	cyto	
*DcINVCW3*	LOC108207625	2	2181	1392	5	4	463	51.52	4.52	25.23	81.21	−0.350	cyto	
*DcINVCW4*	LOC108215200	3	2895	1740	7	6	579	65.67	6.95	37.67	77.27	−0.378	vacu	
*DcINVCW5*	LOC108216856	4	4148	1914	7	6	637	70.93	5.23	36.47	85.40	−0.237	cyto	
*DcINVCW6*	LOC108218455	4	5391	1779	6	5	592	66.72	8.99	28.53	80.35	−0.354	chlo	
*DcINVCW7*	LOC108218454	4	2756	1782	7	6	593	66.54	5.91	25.54	80.86	−0.283	cyto	
*DcINVCW8*	LOC108222818	5	3814	1899	7	6	632	69.99	5.06	31.06	86.36	−0.198	nucl	
*DcINVCW9*	LOC108226467	6	2852	1770	7	6	589	67.11	8.66	32.75	78.76	−0.326	vacu	
*DcINVCW10*	LOC108195234	7	2572	1536	5	4	511	58.02	6.84	38.39	73.62	−0.451	nucl	
*DcINVVR1*	LOC108206477	2	4985	1956	7	6	651	72.71	5.80	35.75	82.46	−0.274	cyto	
*DcINVAN1*	LOC108205019	1	5399	1674	5	4	557	63.63	5.97	49.34	81.10	−0.229	cyto	
*DcINVAN2*	LOC108204816	1	5331	2028	6	5	675	76.10	8.26	37.30	85.75	−0.285	chlo	
*DcINVAN3*	LOC108194115	1	5095	1746	5	4	581	65.69	6.77	47.63	81.14	−0.289	nucl	
*DcINVAN4*	LOC108207261	2	3223	1983	6	5	660	74.78	6.53	39.06	82.27	−0.367	chlo	
*DcINVAN5*	LOC108209016	2	4614	1914	7	6	637	71.65	6.49	44.10	86.23	−0.242	cyto	
*DcINVAN6*	LOC108224631	6	4951	1713	5	4	570	65.31	6.16	58.97	81.46	−0.252	cyto	

Abbreviations: Aa(s), amino acid(s); CDS, coding DNA sequence; Chr, chromosome; pI, isoelectric point; GRAVY, grand average of hydropathicity; nucl, nucleus; chlo, chloroplast; cyto, cytoplasm; vacu, vacuole.

## Data Availability

The original contributions presented in this study are included in the article/Supplementary Material. Further inquiries can be directed to the corresponding authors.

## Supplementary Materials

The following supporting information can be downloaded at https://www.mdpi.com/article/10.3390/genes17101184/s1, Figure S1: Mofit sequence; Table S1: Co-linear relationships and Ka/Ks of *DcSPSs*, *DcSUSs*, *DcINVs*; Table S2: Detailed information of cis-acting elements identified in promoter region of *DcSPSs*, *DcSUSs*, *DcINVs*; Table S3: The TPM values of *DcSPSs*, *DcSUSs*, *DcINVs* in different tissues; Table S4: The Log_2_ (TPM + 1) values of *DcSPSs*, *DcSUSs*, *DcINVs* in different tissues; Table S5: Primers used for qRT-PCR analysis.

## Abbreviations

The following abbreviations are used in this manuscript:

SPS	sucrose phosphate synthase
SUS	sucrose synthase
INV	invertase
UDPG	uridine diphosphate glucose
F6P	fructose-6-phosphate
S6P	sucrose-6-phosphate
SPP	sucrose-6-phosphate phosphatase
UDP	uridine diphosphate
AINVs	acidic invertases
NINVs	alkaline/neutral invertases
Aa	amino acid
CDS	coding DNA sequence
Chr	chromosome
pI	isoelectric point
GRAVY	grand average of hydropathicity
nucl	nucleus
chlo	chloroplast
cyto	cytoplasm
vacuole	vacuole
